# 3D-printed integrative probeheads for magnetic resonance

**DOI:** 10.1038/s41467-020-19711-y

**Published:** 2020-11-13

**Authors:** Junyao Xie, Xueqiu You, Yuqing Huang, Zurong Ni, Xinchang Wang, Xingrui Li, Chaoyong Yang, Dechao Zhang, Hong Chen, Huijun Sun, Zhong Chen

**Affiliations:** 1 Department of Electronic Science, Fujian Provincial Key Laboratory of Plasma and Magnetic Resonance, Xiamen University, 361005 Xiamen, China; 2 State Key Laboratory for Physical Chemistry of Solid Surfaces, Xiamen University, 361005 Xiamen, China; 3 Department of Chemistry, Xiamen University, 361005 Xiamen, China; 4 Pen-Tung Sah Institute of Micro-Nano Science and Technology, Xiamen University, 361005 Xiamen, China; 5 Fujian Science & Technology Innovation Laboratory for Energy Materials of China, 361005 Xiamen, China

**Keywords:** Electrical and electronic engineering, Design, synthesis and processing, NMR spectroscopy

## Abstract

Magnetic resonance (MR) technology has been widely employed in scientific research, clinical diagnosis and geological survey. However, the fabrication of MR radio frequency probeheads still face difficulties in integration, customization and miniaturization. Here, we utilized 3D printing and liquid metal filling techniques to fabricate integrative radio frequency probeheads for MR experiments. The 3D-printed probehead with micrometer precision generally consists of liquid metal coils, customized sample chambers and radio frequency circuit interfaces. We screened different 3D printing materials and optimized the liquid metals by incorporating metal microparticles. The 3D-printed probeheads are capable of performing both routine and nonconventional MR experiments, including in situ electrochemical analysis, in situ reaction monitoring with continues-flow paramagnetic particles and ions separation, and small-sample MR imaging. Due to the flexibility and accuracy of 3D printing techniques, we can accurately obtain complicated coil geometries at the micrometer scale, shortening the fabrication timescale and extending the application scenarios.

## Abstract (web summary)

Here, the authors combine 3D printing and liquid metal filling techniques to fabricate customised probeheads for magnetic resonance experiments. They demonstrate in situ electrochemical nuclear magnetic resonance analysis, reaction monitoring with continues-flow separation and small-sample imaging.

## Introduction

With the extensive development of nuclear magnetic resonance (NMR) and magnetic resonance imaging (MRI) techniques, these methods have found wide applications in various fields, such as oncology imaging, biological material detection, substance analysis, and in situ electrochemical reaction monitoring^1–4^. As one of the core components of magnetic resonance (MR) systems, radio frequency (RF) coils significantly influence the quality of MR experimental results. Conventional MR coils are usually fabricated by manual winding and printed circuit board lithography techniques, which generally require labor-intensive manufacturing and 2D fabrication processes^5–7^. Thus, it is imprecise and time consuming to fabricate coils with complex or irregular 3D structures, especially given demands of miniaturization. Moreover, some unconventional NMR experiments, such as microliter-scale sample detection and biochemical reaction monitoring, require customized 3D microfluidic sample structures integrated with RF coils^8,9^. It is difficult for MRI samples that have different shapes and sizes or microfluidic systems to fit RF homogeneous regions exactly, which leads to a reduction of signal-to-noise ratio (SNR) due to a lower filling factor. To overcome these difficulties, we have developed an integrative MR probehead fabrication method based on the combination of high-precision 3D printing and liquid metal (LM) infusion techniques.

The 3D printing (or additive manufacturing) technique is a process of creating 3D objects with customized shapes and geometries by using different materials^10^. The use of 3D printing instead of subtractive manufacturing methods, such as mechanical machining and laser cutting, has attracted great interest in numerous applications in the field of rapid prototyping^11^. Three-dimensional microstructures created by 3D printing have been shown to have valuable applications in numerous fields, ranging from biomaterials to microelectronics^12,13^. 3D printing enables high flexibility in sample geometrical design, and provides a potential solution for fabricating stereoscopic RF coils with intricate constructions and sample detection regions consistent with RF coils with high geometrical precision. Despite applications in auxiliary detection phantoms and supporting structures, 3D printing techniques still exhibit inherent difficulties in the fabrication of MR probeheads. These difficulties come from the special requirements for probehead structures and materials, including the coil structure-induced filling factor, material electromagnetic performance, and material magnetic susceptibility^14,15^.

We demonstrate an approach, combined with computer-aided manufacturing and design, to fabricate integrative MR probeheads using 3D printing and LM injection techniques. The MR probeheads consist of a RF coil with micrometer-scale conductive wires, customized sample chambers, and RF circuit interfaces, all of which are wrapped in a single 3D-printed polymer block. Custom-built MR coils, fabricated by perfusing channels with LM at room temperature, were integrated with complex sample chamber geometries and microfluidic systems. To the best of our knowledge, although there are some similar applications^16–18^, no previous studies have explored this type of method for the fabrication of integrative probeheads for MR systems. Using this method, we can build customized probeheads with coil structures that are more precisely adapted to sample dimensions and specifications. Thus, our probeheads can not only enhance the SNR due to the improved fill factor, but also meet the requirements for in situ chemical reaction monitoring and small-sized object imaging. The probehead performance can be further improved based on material screening and structural design optimization.

## Results

### Probehead design and fabrication

**Fig. 1:**
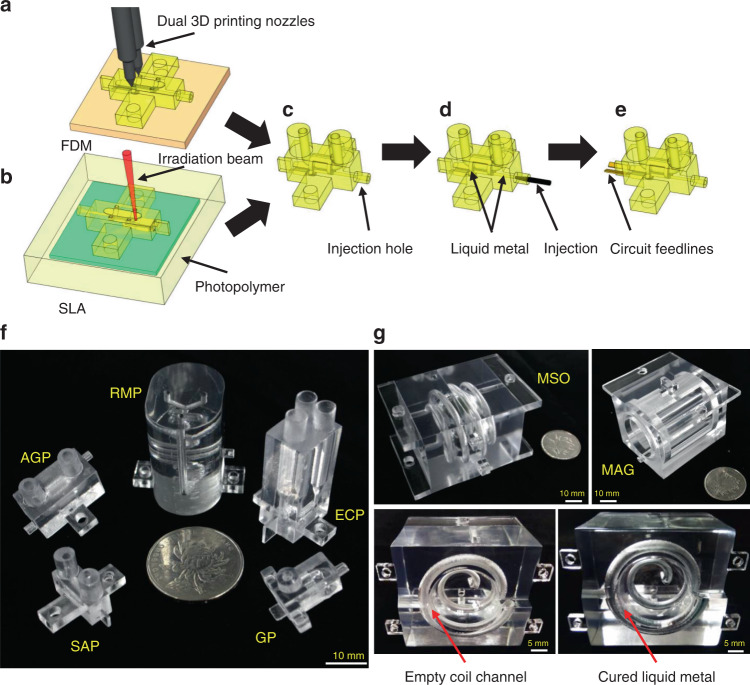
3D printing and manufacturing procedure of integrative MR probeheads for different scenarios. Both **a** fused deposition modeling (FDM) and **b** stereo lithography appearance (SLA) techniques are utilized to fabricate a complete probehead (**c**) layer by layer according to the simulation design. **d** Liquid metal is perfused into the model through the injection hole to form an RF coil. **e** The RF coil is connected to the matching circuit by two copper strips to form a complete probe. The entrance and exit of the liquid metal channel are completely sealed with silver paste. Various 3D-printed probeheads suitable for MR applications can be fabricated and utilized, including **f** U-tube saddle probehead (SAP), U-tube Alderman-Grant probehead (AGP), reaction monitoring probehead (RMP), electrochemical reaction monitoring probehead (ECP), gradient probehead (GP) for MR, and **g** modified solenoid imaging probehead (MSO), modified Alderman-Grant imaging probehead (MAG) for MRI. The coil channel of MSO probehead, before and after the liquid metal perfusion, are also shown.

Various MR coil structures and sample chamber geometries were custom designed in substrate blocks according to the experimental requirements. The dimensions of each coil were determined to fulfill the detection requirements for different sample volumes and MR system conditions. We simulated MR coils by using CST Microwave Studio (CST studio suite 2018, Computer Simulation Technology of America, Framingham, Massachusetts, USA) to determine the optimal RF magnetic field strength and homogeneity. To complete the welding and installation of the probeheads, additional protruding structures were designed. After the design was completed, different parts of the monolithic MR probehead were consolidated in SolidWorks software and fabricated by 3D printing. To inspect the performance of different 3D printing techniques for MR probehead applications, we adopted two of the most commonly used printing principles, i.e., fused deposition modeling (FDM) and stereolithography apparatus (SLA), to fabricate integrative MR RF probehead models. After the probeheads were printed, the necessary postprocessing was performed, and then the conductive coils were constructed using the liquid metal perfusion technique. Figure 1a–e illustrates the printing and manufacturing procedure of our designs.

Multiple 3D-printed integrative RF probeheads for MR were designed (Supplementary Fig. 1) and printed (Fig. 1f, d). All probeheads were printed by using transparent materials to clearly illustrate their internal channel structures. The designed coil structures with different dimensions, such as solenoid, saddle and Alderman-Grant (AG) coils, were embedded inside polymer blocks. A U-tube saddle probehead (SAP) and U-tube Alderman-Grant probehead (AGP) were designed to perform routine microliter-scale sample experiments. To investigate in situ reactions, we designed multiturn microfluidic systems inside in situ reaction monitoring probehead (RMP). The microfluidic channels act as micro-reactors that are integrated with NMR coils for in situ reaction kinetics monitoring with high sensitivity. An electrochemical reaction monitoring probehead (ECP) was fabricated to achieve in situ electrochemical observations. Designed with gradient coils, a gradient probehead (GP) can achieve complex NMR sequence analysis. In addition, a modified solenoid imaging probehead (MSO) and modified Alderman-Grant imaging probehead (MAG) were designed for use in MRI experiments on small samples with specific structural sizes.

### Building materials selection

Both FDM and SLA 3D printers were used to fabricate the RF probeheads with various building materials. Considering the stringent requirements for probehead materials to restrain energy dissipation, magnetic field inhomogeneity and the bottom envelope of the spectra in MR experiments^19^, we characterized the electromagnetic properties, including dielectric constant and loss tangent, of selected building materials in the MR frequency range. In MR RF design, it is essential to use materials with low-dielectric-constant and low-dielectric-loss to decrease the total electromagnetic loss in the signal path, improve coils’ quality factor (*Q*) and the SNR, and thus increase the minimum detectable signal^14^. The analytical results of the dielectric properties of the 3D-printed building materials and unloaded *Q* of SAP probeheads with these materials are provided in Table 1. All *Q* factors were measured on unloaded probes, using an Agilent E5071C network analyzer. Poly tetra fluoroethylene (PTFE) is a reference material that cannot be used for 3D printing, so its *Q* factor is not listed in the table. Modified LM paste (discussed below) is used as conductive material. After comparing the dielectric properties of the materials and corresponding SAP *Q* factors characterized in MR-relevant frequency ranges, we chose PLA (i.e., VisiJet M3 Crystal), which has lower dielectric values in the relevant resonance frequency range, as the probehead building material.

**Table 1:** Dielectric constant and loss tangent of 3D-printed building materials.

	VisiJet M3 Crystal	Clara A	Formula L1	PT-B405	PT-C405	PT-G405	PT-W405	PT-Y405	PTFE (Ref)
*ε*_*r*_	2.68	2.94	2.98	3.28	3.27	3.28	3.27	3.28	2.50
tanδ	0.012	0.016	0.019	0.025	0.029	0.026	0.022	0.027	0.003
*Q*	45	36	33	28	24	27	30	26	–

Unloaded quality factors of LM SAP probehead were also measured with these different materials.

The magnetism of building materials is also an extremely important factor. The components of the building materials we selected are mainly nonmetallic materials, including polylactic acid, acrylonitrile butadiene styrene copolymers, or acrylic resin. These components are all diamagnetic materials and have little effect on MR experiments. In addition, because the RF coils were embedded in 3D-printed substrates, whether the materials contained hydrogen and the position/size of the material spectral bottom envelope were also key considerations in finally choosing a building material. Related onboard tests needed to be performed after the complete probes were fabricated with building materials that showed excellent electromagnetic properties in previous evaluations. The onboard experimental results proved that PLA performed well in terms of both electrical performance and material background signals, so it was the preferred substrate for our 3D printing models. Although the availability of our measurement results is limited due to the commercial confidentiality of the materials, these performance evaluation aspects still hold considerable reference value for building material optimization and selection.

### Conductive coil liquid metals optimization

Low-melting-point LMs were injected as conductive materials into 3D-printed micro-channels to form MR coils. We first considered several commonly used liquid metal pastes. Instead of mercury and silver pastes^20^, we chose gallium (Ga) and its alloys (e.g. EGaIn) as alternative conducting LM materials due to their low toxicity, high conductivity, good liquidity, and low thermal expansion. In addition, when exposed to air, gallium generates a thin (~1 nm) passivating oxide layer on its surface to protect inside the parts from excessive oxidation, thus benefiting the stability of the entire probehead^21^.

**Fig. 2:**
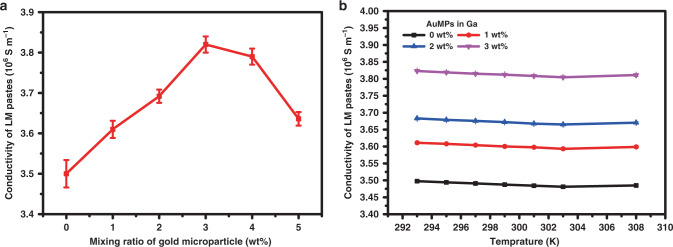
Multi-proportion electrical performance and temperature-dependence characteristic measurement of LM pastes. **a** Conductivity of LM pastes consisting of gold microparticles (AuMPs) and gallium with different mixing ratios. **b** Temperature dependence of the conductivity of gold microparticles in gallium with different mixing ratios. Data represent means ± s.d. from five (**a**) and three (**b**) independent replicates. Source data are provided as a Source Data file.

MR coils should have excellent electrical conductivity to reduce the thermal noise of conductive wires, thus improving the frequency response and quality factor and subsequently increasing the SNR of the acquired spectra. For this reason, the electrical properties (mainly conductivity) of the LMs were important indicators used for measurements. To increase the conductivity of the MR coil materials, we proposed an approach for LM paste preparation by incorporating uncoated metal microparticles into gallium. According to the measurement results (Supplementary Fig. 5), we chose gold microparticles (AuMPs) as mixed metal, as Ga/Au paste with 1 wt% Au microparticles had better electrical conductivity than pure gallium, with the specific conductivity increased by ~3%. We then tested the resistance of Ga/Au pastes with different mixing ratios using I-shaped mold. As the AuMPs content increased, the conductivity of the pastes first increased and then decreased, as shown in Fig. 2a. At an AuMPs ratio of 3 wt%, the conductivity of the LM reached a maximum of 3.82 × 10^6^ S m^−1^, ~10% higher than that of pure gallium. AuMPs dispersed in pure gallium may act as an electronic transmission bridge and thus improve the conductivity of the mixed LM pastes. The reason why the resistance of the Ga/Au pastes unexpectedly increased is presumably related to the processing and molding methods of conductive materials. As the amount of mixing particles increases, the contact resistance (caused by lattice mismatch^22^, etc.) of metal micron-sized particles to LM may increase due to the prolonged mixing time. In addition, the applied pressure during the molding can affect the uniformity of the metal particle distribution in LM pastes, and cause surface micron-level cavities. The above effects were enhanced with the increasement of metal particle content.

The saddle coil electrical conductivity was also measured with respect to temperature to show its performance under long, complex MR pulse sequences, as shown in Fig. 2b. The electrical performance of the LM pastes remained stable over the MR working temperature range (293–308 K), with differences of <5%. We prefer solid-state LMs to be conductive materials in experiments because of their better electromagnetic stability than liquid state LMs. During NMR experiments, we used an air circuit to control the probehead temperature in order to maintain the solid-state stability of the coil. However, LM coils may still experience a partially slightly heat-melting due to the long RF pulse duration, sample reaction heating and other reasons. The solidification rate increases after mixing because the metal particles exist as condensation nucleus in LM, inhibiting the supercooled phenomenon of gallium. This effect improved the stability of the probeheads as a result of the enhanced thermal tolerance of the MR coils.

The magnetic properties of LM pastes are mainly determined by gallium. Pure gallium has a low diamagnetism, −0.248 × 10^−6^ cGSM g^−1^ mass magnetic susceptibility in the solid phase (−0.085 × 10^−6^ cGSM g^−1^ for Gu), and very low paramagnetism, 0.002 × 10^−6^ cGSM g^−1^ mass magnetic susceptibility in the liquid phase^23^. In the experimental temperature range, the magnetic properties of the LM pastes had little effect on our experiments.

### NMR coil simulation and verification

**Fig. 3:**
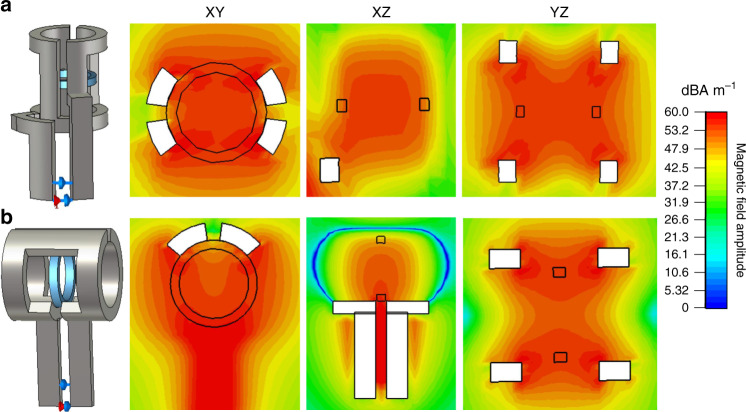
RF magnetic field simulations of saddle and modified Alderman-Grant coils. Simulations of saddle (**a**) and modified Alderman-Grant (**b**) coils were both performed at a frequency of 500 MHz.

Designing and simulating RF coils for MR applications are cumbersome but fundamental tasks, in which the prediction of SNR performance, RF field homogeneity and depth of penetration greatly speeds up the design process by reducing the required hardware manufacturing iterations^24^. According to our integrated probehead design requirements and 3D printing technique limitations, a capacitor could not be included in the coil structure. This meant that all candidate coil structures should be noncapacitive. There are three main types of noncapacitive volumetric coil structures: solenoid, saddle, and modified AG structures. Solenoid coils with excellent performance require a sufficient number of turns, which increases the inherent inductance of the resonant circuit, making it difficult to tune the coil to a high frequency (500 MHz in our design). A high number of turns also increases thermal noise of the resonant circuit, reducing the quality factor and SNR of the coil^14^. Therefore, after determining the building material and conductive metal for the probeheads, we simulated two common noncapacitive coils, saddle and modified AG coils, for applications in a Varian 11.7T NMR system. The homogeneity of the generated RF field in the signal detection area is an important index for evaluating coil experimental performance. For microliter-scale sample detection and miniaturization requirements, an inner diameter of 3 mm and thickness of 400 µm were selected to define coil channel structures that are easy to clean and inject. The coils were designed and modulated based on the optimal sizes^25^; i.e., heights of 4.98 mm and 4.5 mm were adopted for the saddle and modified AG coils at first, with the aim of maintaining a homogeneous field with a height of ~2 mm. To evaluate the optimal performance of each coil, a RF (B_1_) field was simulated using CST Microwave Studio (Fig. 3). The simulation results (with PEC as conductive material) demonstrated that the saddle coil had a higher *Q* (172), better RF field uniformity (*ΔB* = ± 4% $\bar B$) in the sample detection area, and a higher normalized SNR (1.0) than AG coil (*Q* = 111, *ΔB* = ± 8% $\bar B$, normalized SNR = 0.61)^14^. In addition, compared an AG coil, a saddle coil with the same small size would be easier to tune to the desired high resonance frequencies, such as 500 MHz in our design. Thus, a saddle coil was chosen as the optimal structure for our NMR experiments.

### In situ electrochemical monitoring using 3D-printed probehead

**Fig. 4:**
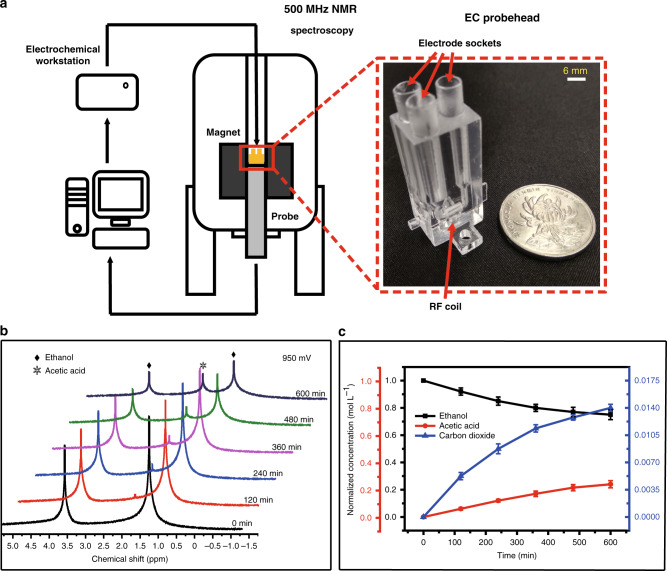
In situ EC-NMR system and experimental results. **a** Schematic representation of the in situ EC-NMR setup and ECP probehead. The in situ ECP probehead contains three customized electrode sockets/channels, which can form a three-electrode structure. **b** In situ ^1^H-NMR spectra and **c** time-resolved changes in the ethanol, acetic acid, and carbon dioxide concentrations during the ethanol oxidation reaction. In **c**, data represent means ± s.d. from three independent experiments. Source data are provided as a Source Data file.

Ethanol offers an attractive alternative as a liquid fuel because of its advantages of hypotoxicity, high energy density and renewability, and its usage considerably reduces dependence on traditional energy sources^26,27^. A 3D-printed electrochemistry-nuclear magnetic resonance (EC-NMR) probehead (i.e., ECP), shown in Fig. 4, was custom designed to perform in situ EC-NMR experiments to investigate the ethanol oxidation reaction. Conventional EC-NMR experiments generally adopt a dedicated reaction chamber. The placement and isolation of multiple electrodes in the tube are complex and time consuming^4,28^. However, such cells can be ignored in our design. Instead of a single sample tube, three long (18 mm) and wide (4.3 mm diameter) sample channels were constructed and integrated with RF coils to insert the electrodes (working electrode Pt, counter electrode Pt, and reference electrode Ag) without creating misconnections and to prevent reaction-generated bubbles from refluxing, which would affect experimental stability. These channels are independent of each other and converge in the NMR signal detection region. To achieve higher electrocatalytic efficiency^29,30^, platinum electrodes incorporated with platinum particles were used as the working and counter electrodes because they are more tolerant to poisoning effect than bulk platinum electrodes due to the adsorption of CO species.

Figure 4a schematically shows the electrochemical probe setup for the in situ EC-NMR study. A series of in situ ^1^H NMR spectra were acquired to monitor the time-dependent information of the products and reactants during the ethanol oxidation reaction at 0.9 V for 10 h using a standard 1D proton NMR pulse sequence, as shown in Fig. 4b. The volume of aqueous solution involved in the reaction was 500 μL and included 1.0 M ethanol as the initial reactant and 0.1 M HClO_4_ as the supporting electrolyte. The NMR peak areas and chemical shifts were calibrated with TSP to classify and quantify the reaction species. Before electrolysis, ethanol showed spectral peaks at 3.57 ppm and 1.08 ppm, corresponding to hydrogen atoms in terminal hydroxymethyl groups (−CH_2_OH) and protons in methyl groups (−CH_3_), respectively. Hydroxyl groups were not detectable due to the fast proton exchange with water. In the progress of electrolysis, a new NMR peak appeared at 2.08 ppm, indicating the formation of acetic acid (CH_3_COOH). The proton peak at 2.08 ppm increased due to the production of acetic acid as ethanol oxidation progressed, while the signals at 1.08 and 3.57 ppm decreased caused by the consumption of ethanol. The main intermediate (acetaldehyde) cannot be observed in the experiment because acetaldehyde polymerization readily occurs under a high oxidation potential, especially when the monomer is adsorbed on active surface sites^26,31^. Thus, acetaldehyde would be further oxidized to acetic acid. The results indicate that the ECP design enables the in situ real-time monitoring of products and provides an efficient tool to gain insight into reaction pathways.

To obtain further insight, quantitative analyses were conducted by integrating ^1^H NMR spectra. Figure 4c shows the changes in the ethanol, acetic acid, and CO_2_ concentrations obtained from NMR measurements by normalizing the peak integrals to the TSP internal reference. Because the main products of ethanol electrolysis oxidation under high potential are acetic acid and CO/CO_2_ gas, one can calculate the quantity of gases from the mass balance. The ethanol concentration decreased significantly in the first 2 h, while the reaction products increased rapidly during the same time period. Then, the reaction rate was reduced to a fairly low level, indicating that the ethanol oxidation activity on the catalyst was probably affected by the blocking of C-C breaking on Pt active sites by intermediate poison species^32^. Incomplete ethanol oxidation to acetic acid thus prevailed over complete oxidation to CO_2_.

### In situ reaction monitoring with continuous flow separation using 3D-printed probehead

Recent years have seen increased interest in the real-time in situ NMR analysis of intermediates and products of chemical reactions^33,34^. The continuous flow separation of sample components is an important step in chemical and biochemical analyses^35,36^. In the detection of samples containing ferromagnetic and paramagnetic particles/ions, NMR spectra may produce the proliferation of spectral lines and spectral peak overlaps due to the uneven local magnetic field, which may lead to difficulties in analyzing experimental spectra in severe cases. Effective particle continuous flow separation methods are essential for in situ NMR, enabling the real-time monitoring of reactions with paramagnetic generators and facilitating the integration of other upstream/downstream control and treatment steps. Several conventional particle/ion separation techniques, commonly employed for industrial and research applications, have significant limitations and difficulties, especially for in situ NMR experiments^33,37^. The implementation of these methods increases instrument complexity and requires additional expensive equipment. We present here a continuous-flow separation probehead (CFSP), without extra equipment to study continuous, in situ reactions in NMR experiments without special treatment in which the reactants or products contain paramagnetic particles/ions affecting normal detection. We utilized the particle size properties and electrical properties of the deposits and ions for separation, taking advantage of the adsorption properties of silica gel and the high magnetic field of NMR magnets. Particle/ion movement in a magnetic field varies greatly depending on these characteristics. Using our probehead, we successfully separated particles and ions with different dimensions and magnetic properties in a natural high-field magnet.

**Fig. 5:**
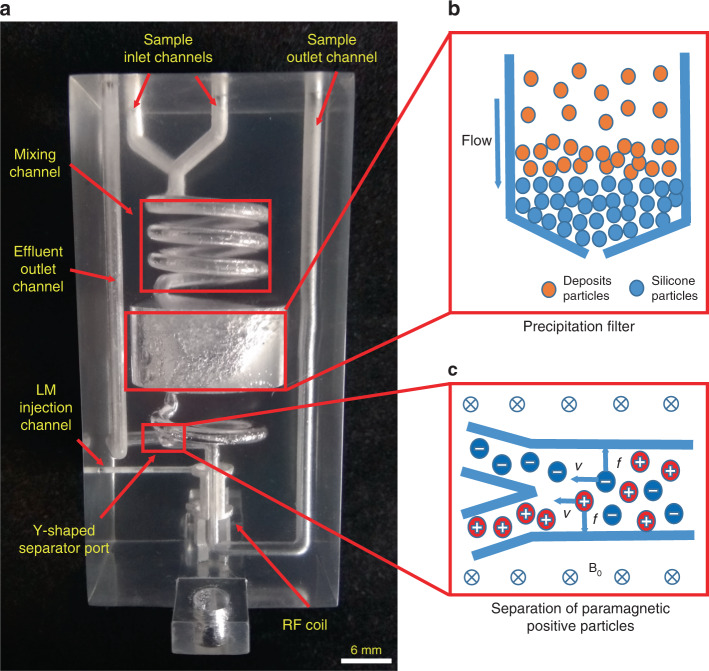
Inner structures and separation principles of the CFSP. **a** The internal structure of the CFSP. **b** The principle of in situ filtration and separation of paramagnetic particles. **c** The principle of the Lorentz force separation of paramagnetic ions under a strong magnetic field.

From Fig. 5a, it can be seen that the particle and ion separation sample channels of the CFSP, a modified version of the RMP, mainly consist of a reaction channel, particle filter channel, ion separation channel and sample/waste liquid shunt channel. The design of the inner channel structure of the probehead is shown in detail in Supplementary Fig. 14. In our experiment, the CFSP was designed to monitor the oxidation reaction procedure of isopropyl alcohol with potassium permanganate in neutral solution, in which the suspended manganese/potassium ion and the produced manganese dioxide particles precipitation needs to be optimally separated from the sample prior to signal detection to prevent a paramagnetic effect in MR experiments^38^. A saddle coil was used in the CFSP to focus the RF field onto the product detection area with a total volume of 13 µL. The two reactants were injected into the probehead through sample inlets and then mixed and reacted in the reaction channel (1.8 mm in channel diameter, 15 mm in spiral diameter, 15 mm in height, 3-turn solenoid). During the reaction, manganese dioxide precipitation was continuously generated and suspended in the solution. As the first separation part of the shunt channel, the adsorption tank (15 mm in height and 25 mm in diameter) of the filter channel was filled with silica gel particles, which have a strong adsorption capability and can effectively filter the particles generated in solution^39,40^, as shown in Fig. 5b. The paramagnetic particles were mainly deposited near the upper surface of the silica gel layer, which was more than 15 mm above the coil detection area and had little effect on the experiment. Silica gel also has a considerable degree of adsorption for metallic paramagnetic ions. Then, the filtered solution entered the ion separation channel (1.8 mm in diameter, 2.5 mm in height, and 1-turn solenoid), in which paramagnetic positive ions (Mn^2+^ and K^+^) were towed by the Lorentz force to the area near the outer wall of the sample channel according to the left-hand rule under the effect of the vertical downward magnetic field, as shown in Fig. 5c. The separation fork consisted of two 45° angled channels at the end of the ion separation channel, and the positive ion-containing portion near the outer wall of the channel was separated into the waste liquid channel for discharge, while the remaining portion with few paramagnetic particles and ions was directed to the detection area for NMR experiments.

The CFD module in COMSOL Multiphysics software was used to simulate the fluid mixing in the spiral channel to demonstrate the mixing efficiency of the two samples. Considering the flow rate of the samples in the microchannel, the laminar flow-dilute concentration substance transfer module was employed for simulation. The mixing of samples in the channel under different conditions can be obtained by scanning parameters such as flow rate, transfer coefficient, and viscosity. The simulation results under the experimental conditions were obtained (Supplementary Fig. 15). The results indicated that the complete mixing of the two reactants in the spiral channel was achieved, even at the highest flow rate (100 μL min^−1^ at the inlet) in our work.

**Fig. 6:**
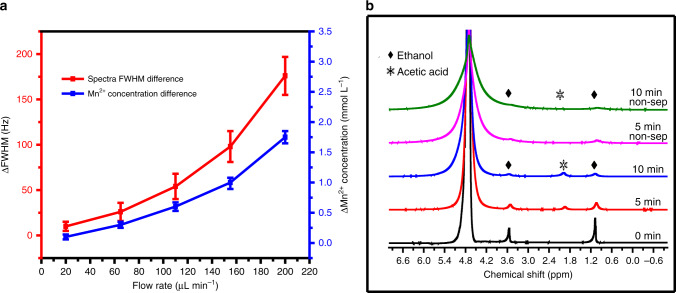
The Mn^2+^ separation efficiency and the in situ separation results of the CFSP. **a** The Lorentz force separation efficiency of paramagnetic ions (0.01 mol L^−1^ manganese ions in aqueous solution) with different flow rates are shown through full-width-at-half-maximum (FWHM). **b** In situ ^1^H-NMR spectra during the ethanol oxidation reaction. With the addition of a filter structure, a spectra containing more useful information can be measured at different reaction times. In **a**, data represent means ± s.d. from three independent experiments. Source data are provided as a Source Data file.

The CFSP probehead (Supplementary Fig. 16) required some preliminary testing and preparation before being used for experiments (Supplementary Methods). We measured the effects of different paramagnetic potassium ion concentrations on the full-width-at-half-maximum (FWHM) of the spectra (Supplementary Fig. 17), which increased sharply with increasing ion concentration, and the rate of increase decreased gradually as the peak intensity approached zero. There was little change in the spectral width of the potassium permanganate solution at different concentrations (Supplementary Fig. 18), indicating that the potassium permanganate solution has little paramagnetism. Therefore, the widen spectra may mainly be derived from the paramagnetic potassium ions and manganese ion impurities in the solution. The actual paramagnetic ion separation efficiency of the probe under high field conditions was also measured in in situ NMR experiments with a 0.01mol L^−1^ manganese ion sample (Fig. 6a). The content of manganese ions in the solution was measured by a manganese ion meter (LH-Mn1, Hangzhou Luheng Biotechnology Co., Ltd., China). There was a significant difference in the paramagnetic ion concentration between the two outlet solutions after separation, which was more clearly demonstrated in the resulting spectra. The Mn^2+^ ion concentration in the effluent outlet channel sample was significantly higher than that in the sample outlet channel sample. The differences in both the spectral FWHM and ion concentration of the two solutions after separation were proportional to the flow rate.

A series of in situ ^1^H NMR spectra, obtained with varying durations of the oxidation reaction, were acquired for 10 min (Fig. 6b). Compared with the results obtained without separation, the FWHM of the spectra of the reactants and products after filtration was significantly reduced, while the SNR and spectral resolution were increased, indicating that paramagnetic particles and ions were removed from the initial product. The spectral line broadening phenomenon still exhibited because the reaction continued after the particles and ions were separated, producing new paramagnetic substances. Gradual changes in the amount of the reactant (ethanol) and product (acetic acid) with increasing residence time can be clearly detected. This result proves that our CFSP probehead not only enables real-time reaction monitoring but also achieves the continuous flow separation of paramagnetic particles and ions, expanding the application field of in situ NMR experiments.

## Discussion

3D-printed integrative MRI probe heads were also custom designed and fabricated to further demonstrate the universal applicability of our approach. Performance tests and experimental results are illustrated in the Supplementary Note 1. Despite the disadvantages in the conductivity of the coil materials, images with higher SNR were obtained using our LM probe. This demonstrated that our approach has great prospects and potential in custom MRI applications, mainly including flexible MRI^41^, integrated MRI, and micro-MRI^42^.

The *Q* factor is one of the most significant performance parameters of the MR probe (coil) and plays a decisive role in signal sensitivity. The *Q* factor of the RF coil is affected by its geometrical structure, material conductivity, and the resonance frequency. The calculation formula for the inherent *Q* factor of the coil is *Q* = *Lω*_*0*_/*R*, where *L* denotes the coil inductance, *ω*_*0*_ represents the angular frequency of the signal, and *R* refers to the coil resistance^14^. The main factors affecting *R*-value are the material conductivity of the coil, coil size, and the skin effect. It is worth mentioning that the skin effect of high-frequency alternating current has a great influence on the actual resistance of RF coils^43^. At a frequency of 500 MHz, the pathway for current flow inside an LM paste conductor is reduced to approximately 2 × *δ* due to the skin effect. The skin depth *δ* for gallium is 19 µm, calculated by the following equation:

1 $$\delta = \sqrt {\frac{\rho }{{\pi f\mu _0}}}$$

where *ρ* is the specific resistivity of gallium, *µ*_*0*_ is the permeability of vacuum, and *f* is the resonance frequency of the coil. Although gallium has ~15 times higher resistance at 0 Hz, copper has an approximately four times smaller skin depth than gallium at 500 MHz due to the skin effect, which decreases the resistive loss of gallium relative to that of copper by a factor of 4.

One way to measure the *Q* factor of the MR probe is to evaluate the ratio of the center frequency *ω*_*0*_ to the 3 dB bandwidth *Δω* of the reflection coefficient (S_11_). The most commonly used formula is *Q* = *ω*_*0*_/*Δω*, which is also used in our work to measure and evaluate the *Q* factor. The *Q* factor of MR coils fabricated by conventional materials (copper or copper alloy) at room temperature can usually reach 100–200, while the coils with special materials in custom applications may have a worse *Q* factor. The performance of the standard or homemade MR probes is presented in Supplementary Table 1^42,44–47^. Among them, the standard Varian commercial probe (slot tube type copper coil) used in our laboratory has a measured unloaded *Q* factor of 158; a solenoid NMR microcoil wrapped with polyurethane-coated copper wires has a *Q* factor of 26 at 300 MHz^46^; an inkjet printing MRI surface coil with silver paste material has a *Q* value of 12.5 at 400 MHz^47^. Despite the advantages of the skin effect, our LM coils (*Q* = 44) perform worse compared to copper coil probes (175) with the same structure and size due to their disadvantage in material conductivity (Supplementary Fig. 19). Although our LM probes are inferior to commercial probes in performance (*Q* factor), they are able to meet the requirements of our customized MR experiments. The applicability of the customized 3D LM probes was verified by the results of various applications in our work. Our design and fabricate approach of the 3D-printed integrative MR probehead focuses on addressing the precise and integrated custom machining problems encountered by conventional methods, rather than optimizing the performance of the probe. Micrometer-scale 3D RF coils and closely compatible sample channels with complex structures can be constructed by this approach flexibly and precisely, according to the requirements of MR experiments. This benefit dramatically improves the usability of our customized probes in special MR experiments.

The performance of our probes still has a lot of space for improvement. Specifically, the coil resistance would be effectively reduced by further optimizing the coil conductive material (such as mixing silver nanowires into LM^48^), contributing to the improvement of the *Q* factor of our probes. Besides, the performance of the probe could also be enhanced by the improvements in the post-processing of the probeheads, including the cleaning and filling of the channels. Moreover, combined with MR experimental requirements, it is necessary to determine an optimal coil structure and size to obtain an MR probe with a high *Q* factor. In the future, state-of-the-art 3D metal printing capabilities may overcome problems such as printing pores, 3D structural accuracy and associativity with other materials, eliminating the need to add conductive components (e.g., copper foil), and capacitors in MR coils^41,49^. It is worth looking forward to, a variety of 3D printing methods and material innovations will further enhance the performance and greatly expand the application prospects of our customized integrative probeheads.

In conclusion, we have demonstrated an approach for the design and fabrication of integrative MR probeheads with high fabrication precision based on the combination of 3D printing and liquid metal filling techniques. Multi-size/multi-shape integrated MR Probeheads containing customized sample channels were rapidly constructed for evaluation and experiments, benefiting from the great flexibility and convenience of our method. The probehead materials, including 3D printing building materials and liquid metal pastes, were characterized, selected and optimized to improve the experimental performance, which also guided us to optimize the MR probehead structures. To further verify the usability of our designs in conventional MR systems, we performed related NMR and MRI experiments using customized 3D-printed probeheads. According to the results, our proposed method is flexible and effectively meets the requirements of MR experiments, thus expanding practical applications. Our ongoing efforts to further improve probehead performance include optimizing the material performance, improving the design structures, and expanding the application areas. The proposed method presents a basis for customized probeheads for NMR studies and clinical MRI detection, opening up a new class of applications in MR systems.

## Methods

### 3D printing and general fabrication process of printed probeheads

Based upon the FDM technique (Fig. 1a), a 3D printing machine (ProJet 3510SD, 3D Systems Inc., Rock Hill, SC, USA) was used to construct MR prototypes with a printing resolution of 30 µm. During printing, the building materials and sacrificial materials were heated and ejected from one of the dual nozzles to construct the molding structures and the coil/sample hollow cavities, respectively. Similar to FDM, SLA (Cyclone W-1, Jiaxing Shanwei Electrical and Mechanical Co., Ltd., China and SLA300 DLC, Weinstein (Xiamen) Industrial Co., Ltd., China) was also used to create probehead models in a layer by layer fashion with a printing resolution of 25 µm based on a process of photopolymerization (Fig. 1b). With the help of CAD, an ultraviolet (UV) laser was focused on a vat of photopolymer resin, causing chains of molecules link to form polymers, which made up the bodies of the probeheads.

After the 3D printing procedure, we infused LM pastes into the coil channels from the injection hole to form conductive electrical MR coils (Fig. 1c, d). Two thin copper strips were inserted into the LM through the coil pins as feedlines to the matching network (Fig. 1e). Then, the injection holes and RF circuit interfaces were sealed with silver paste (Pelco 16040-30, Ted Pella Inc., Redding, CA, USA) and epoxy glue to prevent LM paste leakage. Because of their strong fluidity, LM pastes can be easily injected into and fully populated in irregular and narrow channels (diameter of 400 µm or smaller in our experiments). An ultrasonic vibration machine (KQ3200DA, Kunshan Ultrasound Instrument Co., Ltd., China) was used to remove tiny air bubbles remaining in the coil channels after LM paste injection. The solidification process, a step used to cure LM pastes to form solid structures, was essential to stabilize the encapsulant. Finally, the inserted RF circuit elements were welded with a matching circuit to constitute a complete MR probe.

### Electrical performance measurement of building materials and LM pastes

Numerous measurement techniques can be used to characterize the electromagnetic properties of 3D printing building materials. We utilized a simple but robust method^19^ to measure the dielectric properties of the selected common building materials used in either FDM or SLA 3D printing (Supplementary Fig. 2). The materials can be broadly divided into three groups, corresponding to three printers: VisiJet M3 Crystal for ProJet 3510SD, Formula L1, and Clara A for SLA300 DLC, and PT-series for Cyclone W-1. The dielectric constant *ε*_*r*_ and loss tangent tan*δ* of the materials in each resonator circuit at MR resonance frequencies were estimated (Supplementary Methods). We fabricated five samples of each building material to minimize the influence of printing inaccuracies, connectors, and parasitic losses on the measurement. Electrical performance tests of the metal materials were carried out in a temperature-controlled chamber. All presented numerical measurements in our work were conducted five times under the same condition to obtain an average.

### Fabrication of LM pastes

LM pastes were prepared by incorporating uncoated metal microparticles into gallium, using high-energy sonication (FS-450N, Shanghai Sonxi Ultrasonic Instrument Co., Ltd., China; Supplementary Fig. 4). The gallium was acid treated to remove the oxide skin, benefiting the incorporation of metal particles into the gallium droplets. Instead of being purely on the exterior of the material, gallium oxide was distributed inside the bulk fluid after sonication^21^. The dispersed oxides served as a support to suspend the metal microparticles in the low-viscosity fluid to form a paste, preventing settling during storage. After mixing and molding, the LM pastes were put aside overnight for stabilization and solidification at 263 K before characterization. The AgMPs and AuMPs (Shanghai Aladdin Biochemical Technology Co., Ltd., China) used for mixing both had a diameter of 10 µm. During mixing, the sonication energy was kept constant, while the amount of microparticles was increased. The AuMPs were weighed using a high-precision electronic balance and mixed with pure gallium at 0, 1, 2, 3, 4, and 5 wt% (of a 2 g mixture).

### Verification tests of the 3D-printed NMR Probehead

To initially verify the performance of our approach, the designed saddle coil structure was imported into SolidWorks software after CST simulation to model the 3D-printed integrative RF probehead. According to the simulation results, an RF coil with a length of 4.98 mm and an inner diameter of 3 mm was finally constructed in a substrate block. To obtain the optimal SNR performance of the coil, we designed the detection region of a sample-filled chamber (2.6 mm diameter) according to the coil geometry (3 mm diameter). The accordingly defined SAP probehead was fabricated as an example with a dimension of 15 mm × 6 mm × 7 mm, and contained specially designed structures for connection and installation (Supplementary Fig. 7). An antenna test, *Q* factor calculation and nutation experiment were performed to test the basic performance of the SAP probehead after welding and installation (Supplementary Figs. 8–10). Because the structure and size of the coil are the same (a preferred saddle coil with optimal dimension scale), the unloaded *Q* factors of the 3D-printed NMR probeheads used in our work are not much different, ranging from 45 to 50. A deionized water signal was acquired using the SAP probehead in a Varian 11.7 T NMR system to demonstrate the usability of our integrated design. The FWHM of the experimental spectrum acquired by using a proton pulse sequence in a single scan was 26 Hz with rough manual shimming.

### Electrode pretreatment and instrument installation in the EC-NMR experiment

Platinum particles were electroplated on platinum wires by cyclic voltammetry in a conventional three-electrode electrochemical cell on a CHI760e workstation (Shanghai CHI Instrument Co. Ltd., China) using the two as-prepared platinum wires as the working electrode and counter electrode and a saturated calomel electrode as the reference electrode at −0.2 V (vs SCE)^29^. The solution consisted of 3.0 mM H_2_PtCl_6_ and 0.1 M H_2_SO_4_. Electrochemical measurements were then performed in a solution of 0.1 M H_2_SO_4_ and 0.1 M C_2_H_5_OH using cyclic voltammetry and chronoamperometry (Supplementary Fig. 12). All chemical reagents, including ethanol (C_2_H_5_OH), chloroplatinic acid hydrate (H_2_PtCl_6_•6H_2_O), sulfuric acid (H_2_SO_4_, 98%), sodium phosphate (TSP) and deuterium oxide (D_2_O, 99.9%), were purchased from Sigma-Aldrich.

The probe with the three-electrode electrochemical probehead (Supplementary Fig. 13) was mounted in the 11.7T NMR instrument. The three electrodes were connected to the electrochemical workstation by copper wire cables, with the electrochemical workstation located approximately five meters away from the magnet to prevent the effect of strong magnetic fields. The electrochemical probehead consisted of a Pt wire as the CE, an Ag/AgCl wire as the RE, and a Pt wire loaded with Pt nanoparticles as the WE. All electrodes were inserted into the sample channels of the customized probehead and sealed with perforated tube caps. The distance between the electrodes and detection area was suitably arranged to not only realize the in situ monitoring of electrochemical reactions, but also effectively avoid the interference of small bubbles generated by the reaction in the monitoring area. The parameters of the conducted proton pulse sequence were a 10 μs excitation pulse length, 57 dBm RF power, 2 s delay, 3 s acquisition time, and 8 accumulations.

### CFSP preprocessing and experiments

A series of pretreatments were required for the CFSP probe prior to NMR experiments. We first used an air pump to blow several small cotton balls into the filter cavity. Due to the funnel-shaped design at the bottom of the filter cavity, the cotton balls gathered at the exit. Silica gel (Shanghai Aladdin Biochemical Technology Co., Ltd., China) with a particle sizes of 250–830 µm were then injected in the form of a deionized water suspension and accumulated in the filtration chamber due to their own stickiness and the blockage provided by the cotton balls. The inner diameter of the channel at the outlet of the filter cavity was reduced to 0.8 mm so that the cotton balls and silica gel particles could be kept in the cavity without being flushed out by the solvent. The probehead was placed for a period of time in a high-temperature drying tank and was used for experiments after the water was evaporated and the silica gel particles were completely dry. Our probehead filtration chamber could hold a 15 mm thickness silica gel layer, ensuring the filtration efficiency of precipitation in experiments.

For in situ NMR reaction monitoring, how to optimize the flow rate of the syringe pump to obtain the best detection results with different durations is extremely complex and important. For a microfluidic system, the channel volume between the mixing point and detecting area and the flow rate are two important parameters that determine the residence time^33^. In our design, we fixed the volume of the mixing channel to 400 µL and precisely adjusted and controlled the flow rate of the reactants through syringe pumps for the detection of products. The flow rate determined the residence time in the channel between the mixing point and detection area, and this residence time was taken as the reaction time. Moreover, the time for fluid to flow through the NMR detection region should also be considered to acquire complete NMR signals with high time resolution. As a result, the flow rate for each syringe pump was set to values from 20 µL min^−1^ to 100 µL min^−1^, corresponding to residence times from 10 min down to 2 min in the reaction channel, and detectable time from 21 s down to 4 s with an estimated detection volume of 14 µL. Samples were injected into the CFSP channels with such a slow flow rate and micropressure, ensuring sufficient time for the silica gel layer to block and adsorb paramagnetic precipitation.

Reactants were pumped into probehead by syringe pumps approximately 3 m outside the NMR magnet (Supplementary Fig. 20). Regular proton pulse sequences were used to detect the spectra of reaction generators after in situ shunting. The delay time between scans was scaled with the flow rate to ensure complete refreshment of the detection volume. The other sequence parameters were consistent with the previous EC experiment. For comparison, the same reaction was detected without filtration using 5 mm sample tube commercial probe (Varian 500 ID/PFG SP50P54TOL), with unloaded *Q* factor equals to 160.

## Supplementary information

Supplementary information

## Data Availability

The authors declare that the main data supporting the findings of this study are available within the article and its Supplementary Information files. Extra data are available from the corresponding author upon request. Source data are provided with this paper.

## Source data

Source data
