# Supplementary material for: 3D-printed integrative probeheads for magnetic resonance
Source: Nat Commun. 2020 Nov 13;11:5793. doi: 10.1038/s41467-020-19711-y (PMC7666178; doi:10.1038/s41467-020-19711-y)
Supplement: Supplementary file 1 — Supplementary information [file 41467_2020_19711_MOESM1_ESM.pdf]

Supplementary Information

3D-printed integrative probehead for magnetic resonance

Authors:
Junyao Xie[1,2], Xueqiu You[1,2]*, Yuqing Huang[1,2], Zurong Ni[1,2], Xinchang Wang[1,2], Xingrui Li[2,3],Chaoyong Yang[2,3], Dechao Zhang[1,2], Hong Chen[3], Huijun Sun[1,2]*, Zhong Chen[1,2,5]*

Affiliations:
1. Department of Electronic Science, Fujian Provincial Key Laboratory of Plasma and Magnetic Resonance, Xiamen University, Xiamen 361005, China
2. State Key Laboratory for Physical Chemistry of Solid Surfaces, Xiamen University, Xiamen 361005, China
3. Department of Chemistry, Xiamen University, Xiamen 361005, China
4. Pen-Tung Sah Institute of Micro-Nano Science and Technology, Xiamen University, Xiamen 361005, China
5. Fujian Science & Technology Innovation Laboratory for Energy Materials of China, Xiamen 361005, China

Correspondence: Zhong Chen (chenz@xmu.edu.cn), Xueqiu You (youxueqiu@xmu.edu.cn), Huijun Sun (sunhj@xmu.edu.cn)

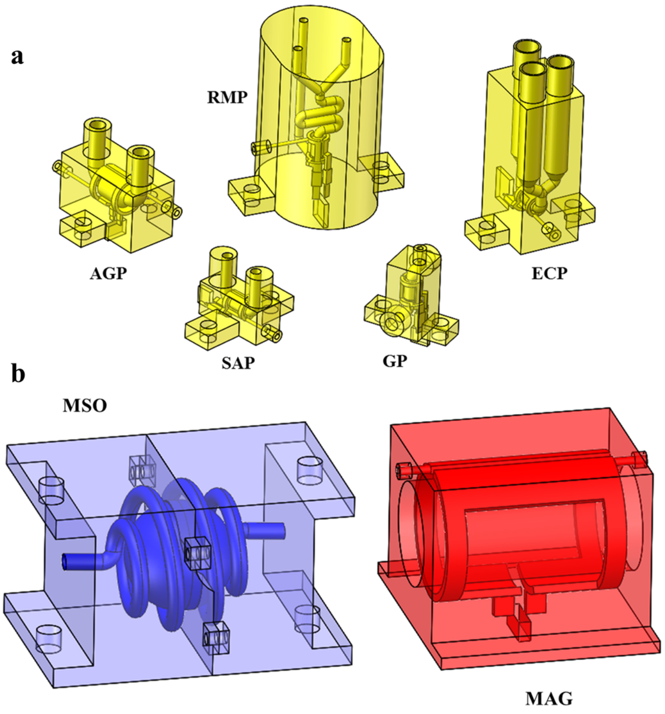

Supplementary Figure 1: The 3D-printed integrative probehead designs. a Various probehead designs suitable for NMR applications, including U-tube saddle probehead (SAP), U-tube Alderman-Grant probehead (AGP), in situ reaction monitoring probehead (RMP), electrochemical reaction monitoring probehead (ECP) and gradient probehead (GP). **b** MRI probehead designs include the modified solenoid imaging probehead (MSO) and modified Alderman-Grant imaging probehead (MAG). Sample detection areas in different probeheads are customized according to RF coil configurations and sample testing requirements.

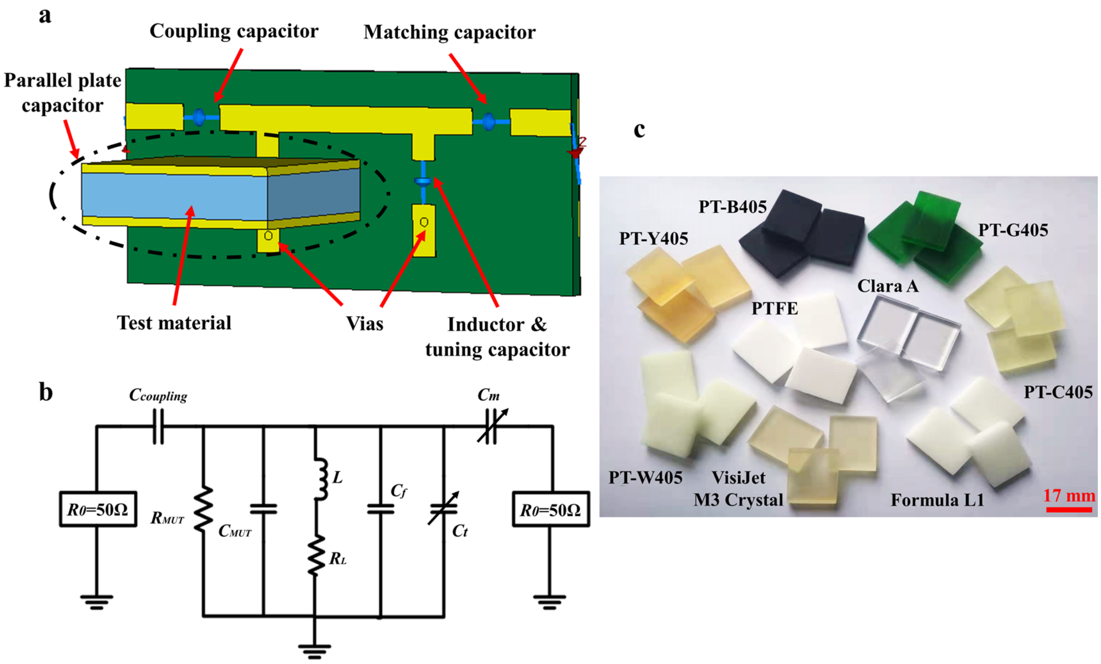

Supplementary Figure 2: 3D printing building material testbench and materials under test. **a** We simulated and built a substrate testbench based on the principle of LC circuit resonance. **b** Tank circuit of substrate testbench. **c** Standard substrates (PTFE) and various 3D printing building materials to be tested.

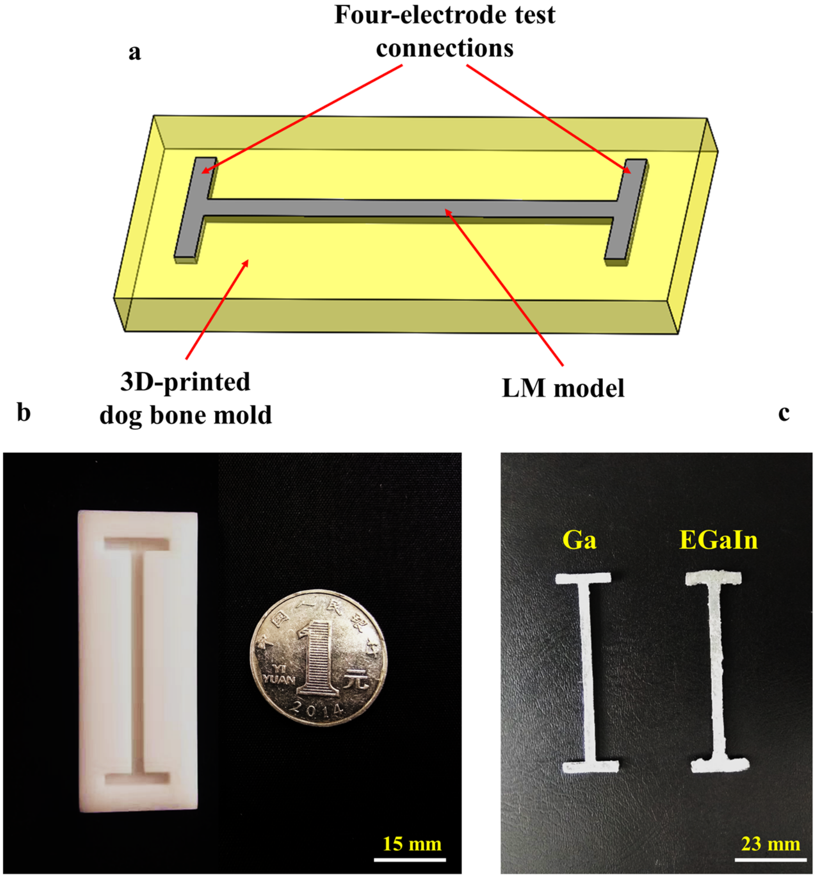

Supplementary Figure 3: Metal mold design and model processing. **a** Liquid metal (LM) material dog-bone model-making mold. **b** Material object of dog-bone mold. **c** Conventional liquid metal models. The model is designed to be slender to show the resistance difference more clearly.

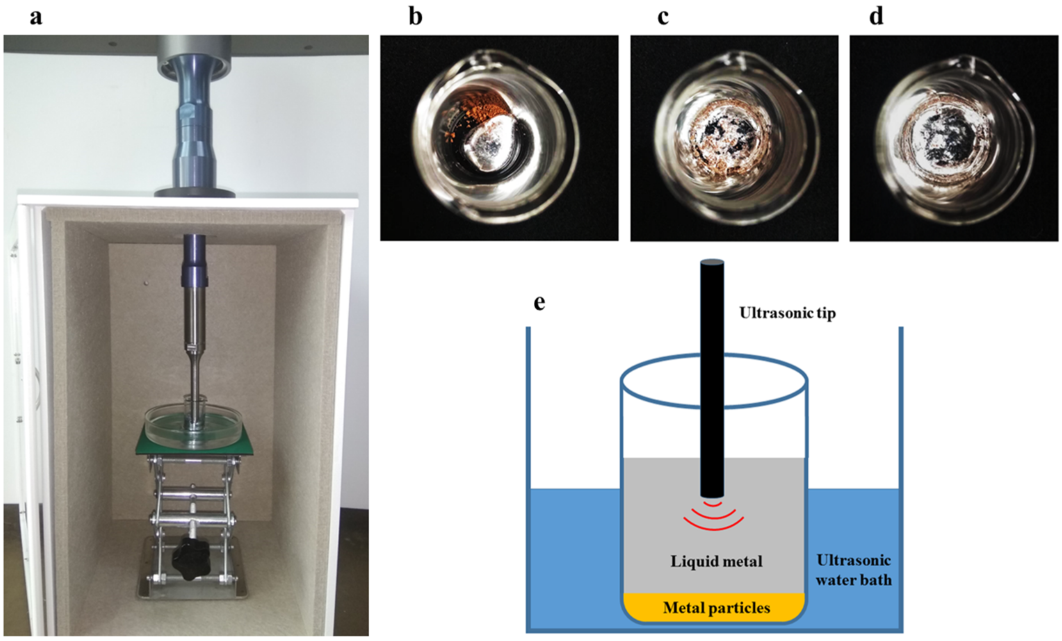

Supplementary Figure 4: The liquid metal pastes preparation method. a Liquid metal and metallic microparticles are placed proportionally in containers and mixed with oscillations using an ultrasonic probe device. **b-d** The mixing of liquid metals and metallic microparticles at different time. **e** Water bath ultrasonic mixing schematic.

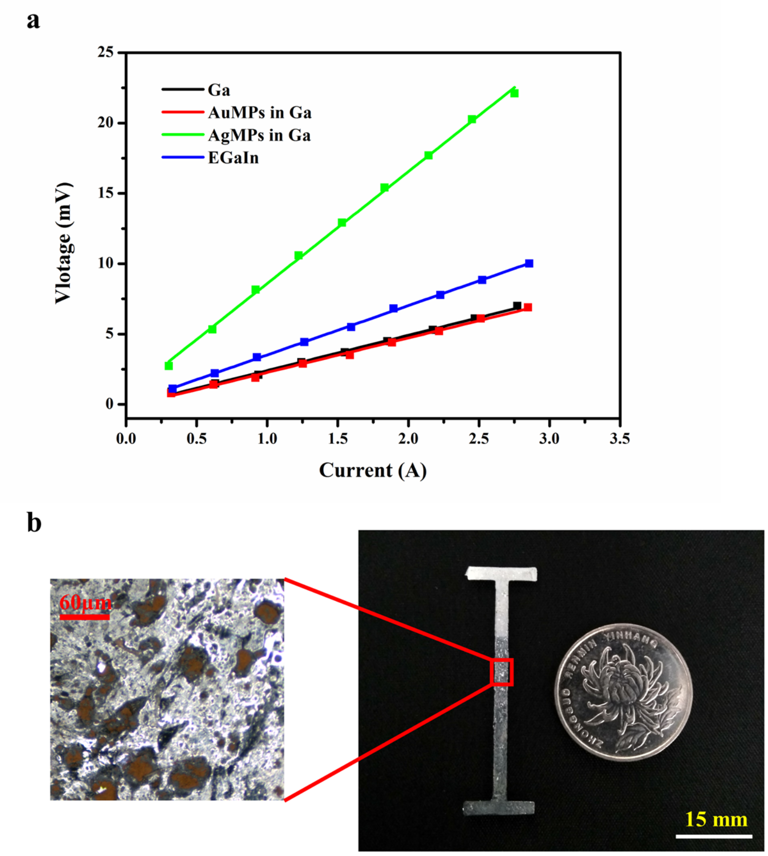

Supplementary Figure 5: DC I-V curves of conductive properties of different LM

pastes and micrograph of the Ga/Au model surface. a Resistance test results of different liquid metal pastes with 1 % metal micro-particles. When about 1 wt% of the gold micro-particle (AuMPs) is added, the resistance drops by approximately 2 %. With the addition of silver micro-particle (AgMPs), the resistance changes abnormally increased, possibly caused by factors such as silver particles stirring oxidation. **b** Material object and micrograph of Ga/Au liquid metal pastes. Micrograph shows the surface of the Ga/Au paste which is observed and measured on an I-shape sample. Source data are provided as a Source Data file.

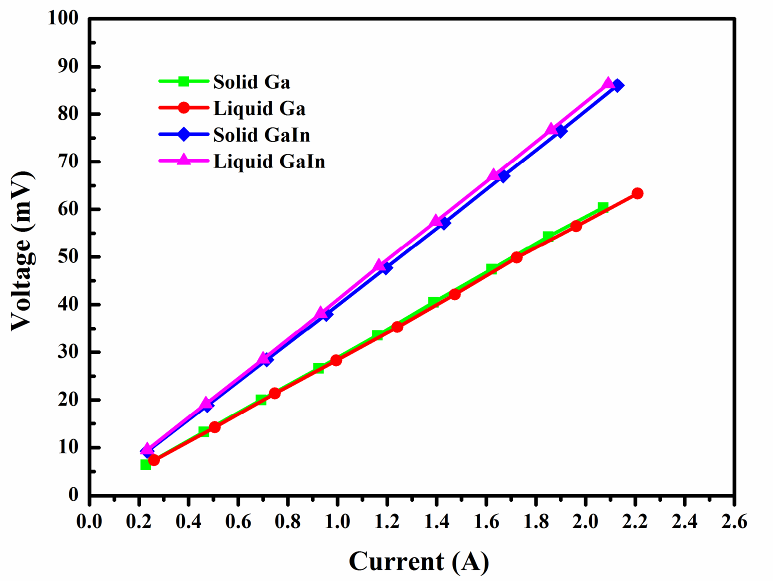

Supplementary Figure 6: The resistance of two conventional liquid metals in solid liquid. The resistance changes of both two liquid metals are less than 5 %. Source data are provided as a Source Data file.

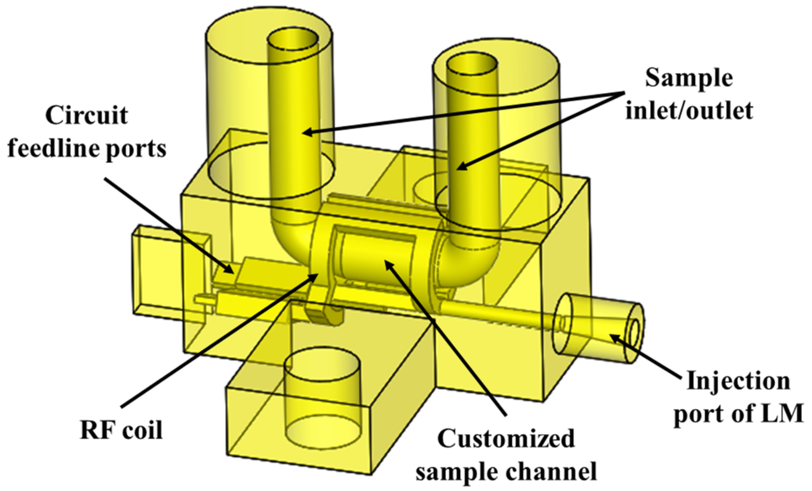

Supplementary Figure 7: An example of the complete 3D printed MR probehead, saddle probehead. The interior of SAP mainly consists of two separate channels, RF coil channel and sample channel. The RF coil channel contains a liquid metal

injection channel, the main body of RF coil, and a circuit connection channel. The sample channel consists of inlet/outlet channels and a customized sample channel.

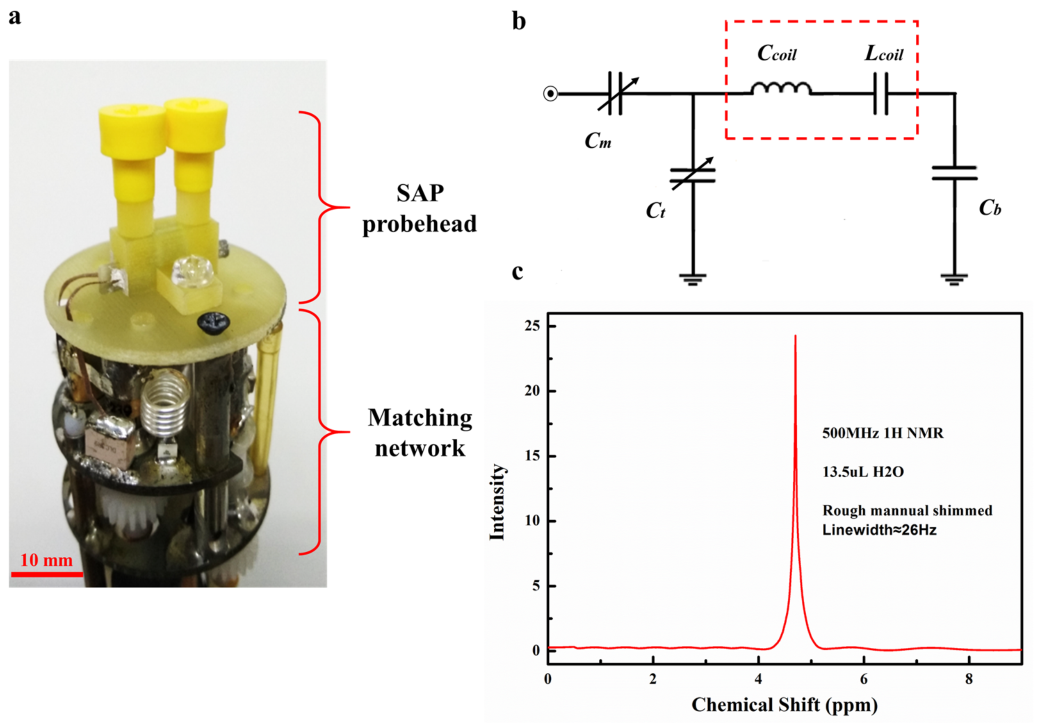

Supplementary Figure 8: The SAP and its experimental performance. a The probe construction of SAP probe, including SAP probehead and matching network. **b** Tank circuit of a saddle coil used in the SAP probe. **c** On-board probe test results with water sample. The balanced matching network was used to tune the resonance frequency to 500 MHz. The circuit is also balanced with an appropriate chip capacitor such that

$$C_{match} + C_{tune} \approx C_{balance} \tag{1}$$

to evenly distribute power and reduce arcing.

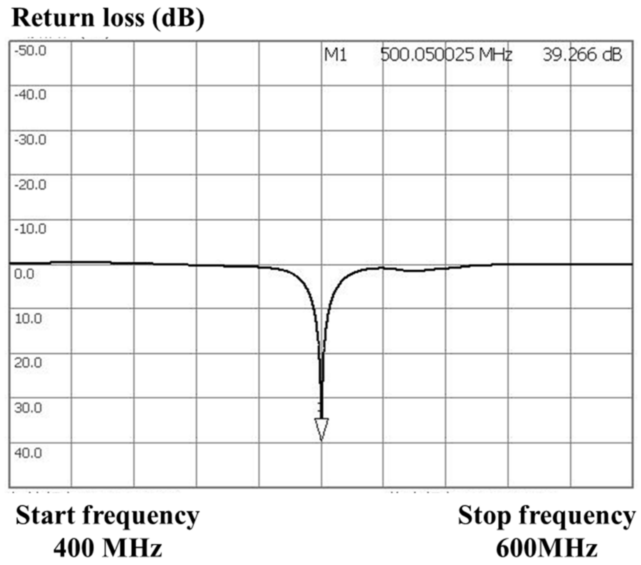

Supplementary Figure 9: Antenna test and *Q* factor calculation of the SAP probe. Antenna test and *Q* factor calculation of the probe were performed on an AV3680A feeder line tester (The 41st Institute of China Electronics Technology Group Corp., China).

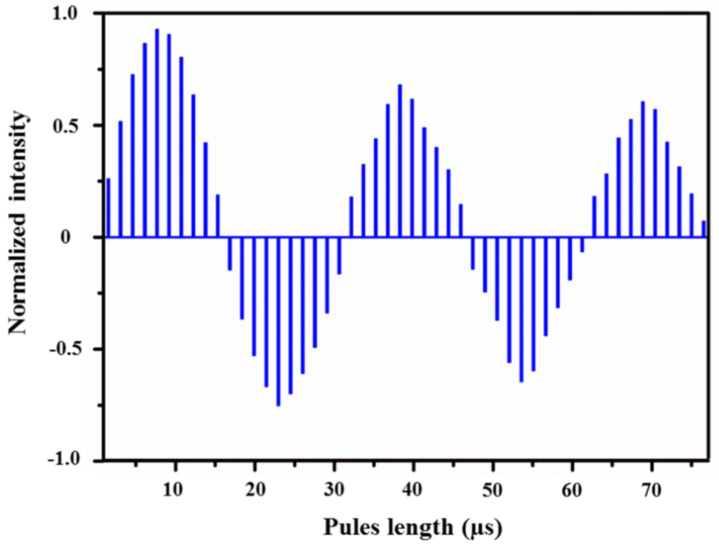

Supplementary Figure 10: Experimental B_1 field homogeneity tests of SAP probe. Nutation experiments were performed with deionized water to detect the spatial uniformity of RF field. A recycle delay of 2 s was used to ensure that the magnetization reached equilibrium. The result shows the plots of the ^{1}H signal as a function of nutation angle acquired using the maximum power of a 100 W RF amplifier. The 90° and 810° flip angles could be achieved by selecting the pulse length and number of experiments. Source data are provided as a Source Data file.

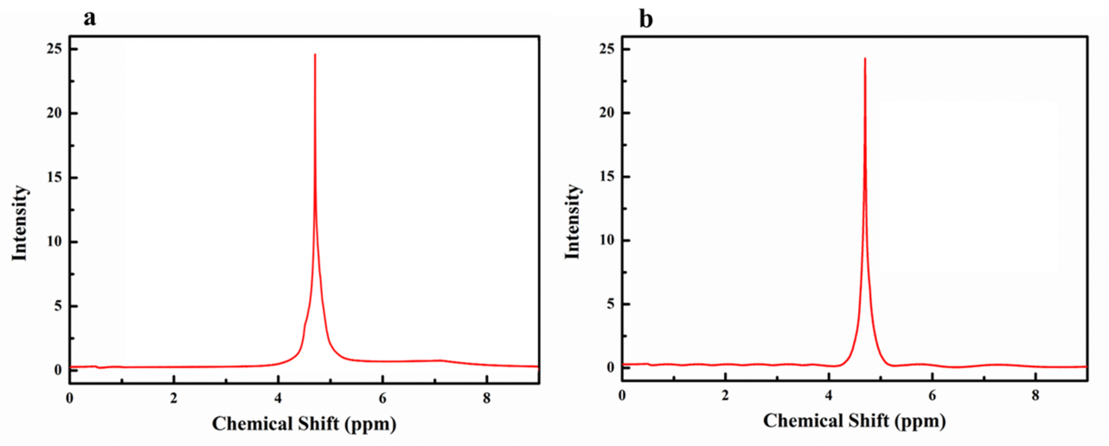

Supplementary Figure 11: Long-term stability experiment of the SAP probe. The results of a single pulse sequence experiments on the water sample when the probe is just ready (**a**) and one year later (**b**). The FWHM of spectra is 26 Hz and 29 Hz, respectively.

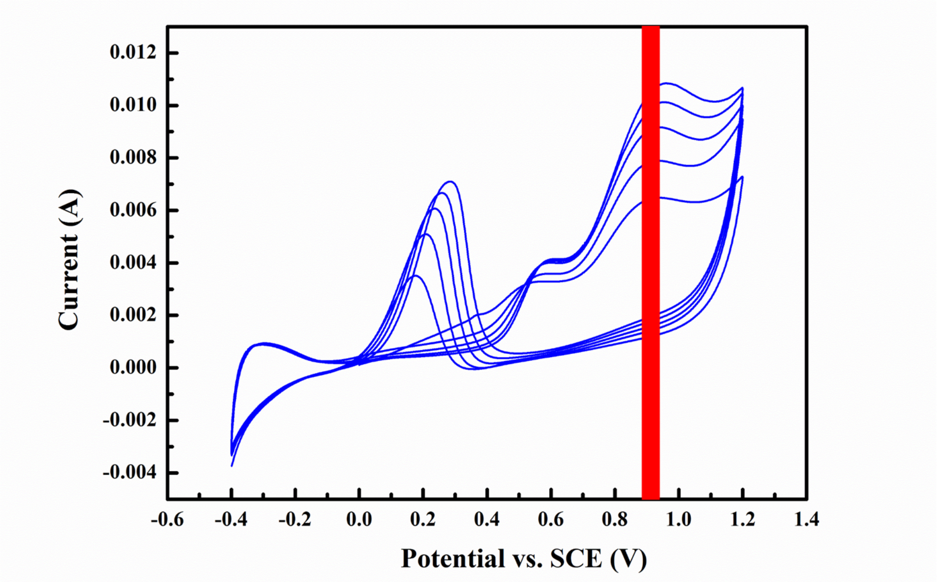

Supplementary Figure 12: CV curves of ethanol electro catalytic oxidation. CV curves were recorded to estimate the electro-catalytic activity of platinum electrode for EOR at a scan rate of 50 mV s^-1 in a potential between −0.2 V and 1.15 V (vs. SCE), followed by the CA tests at 0.85 V (vs. SCE) for 4 hours to evaluate the stability.

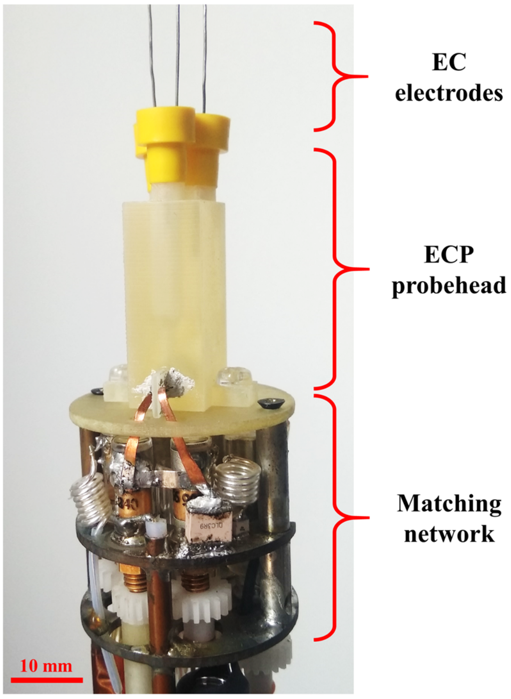

Supplementary Figure 13: Probe construction of ECP probehead. The ECP probe mainly consists of EC electrode interfaces, ECP Probehead, matching network and probehead support.

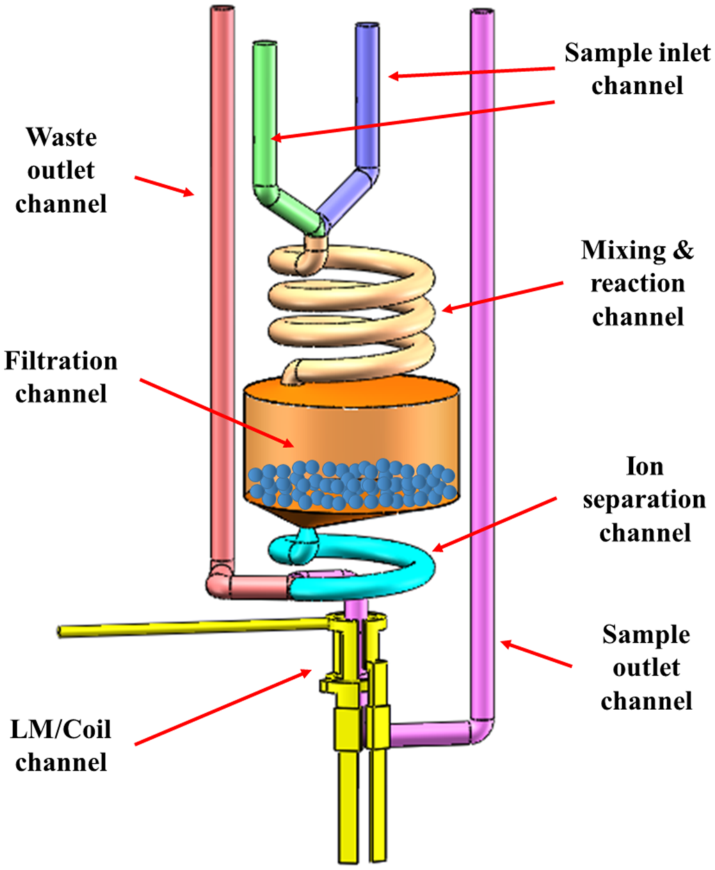

Supplementary Figure 14: Structure design of *in situ* reaction and separation channels of CFSP probehead. The magnetic particles and ions produced by the reaction are excluded from the detected species by adsorption filtration and the separation of Lorenz forces.

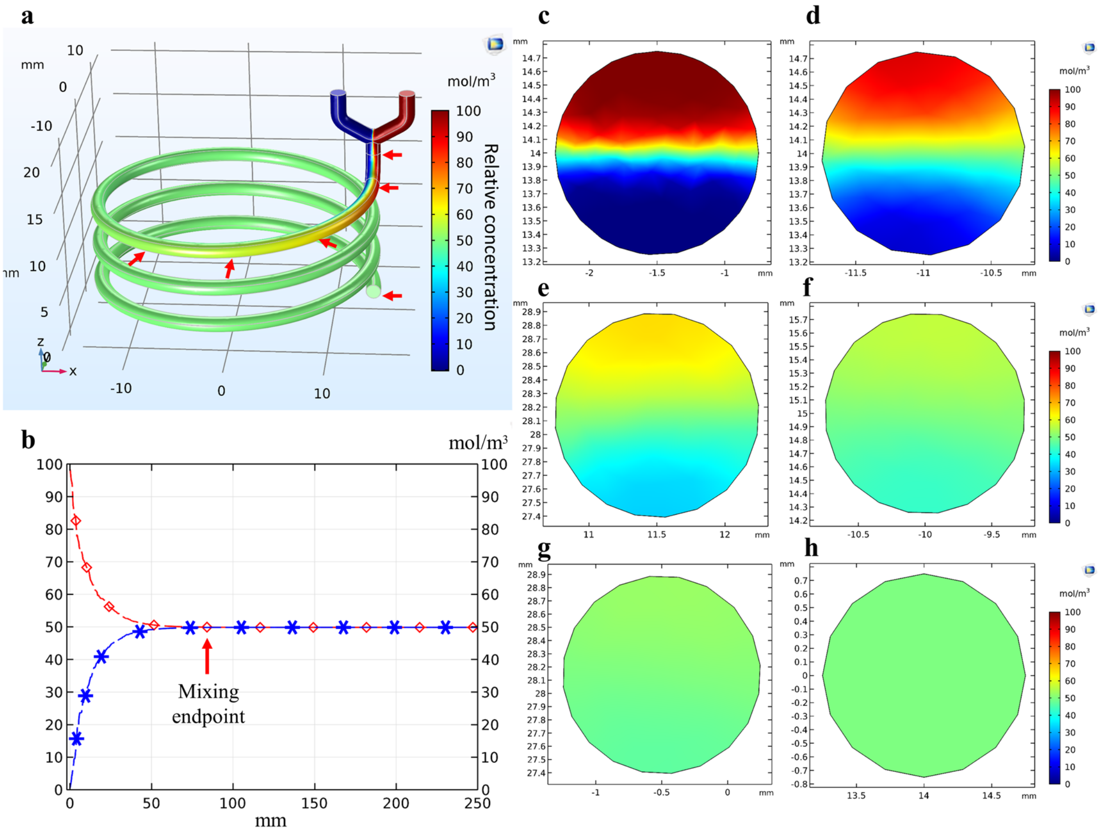

Supplementary Figure 15: CFD simulation results of the sample mixing in spiral channel. a The 3D schematic diagram shows the concentration of the two reactants on the surface of the channel. The rainbow colors represent the reactant concentration. **b** The two-dimensional diagram of concentration in channel shows that a reactant diffuses laterally with the extension of the channel, and finally achieves complete mixing at 86 mm. **c-h** The cross-sectional views of different parts of the channel. The mixing state of different cross sections reveals the mixing rate in the channel. The corresponding positions are marked sequentially in **a**. Source data are provided as a Source Data file.

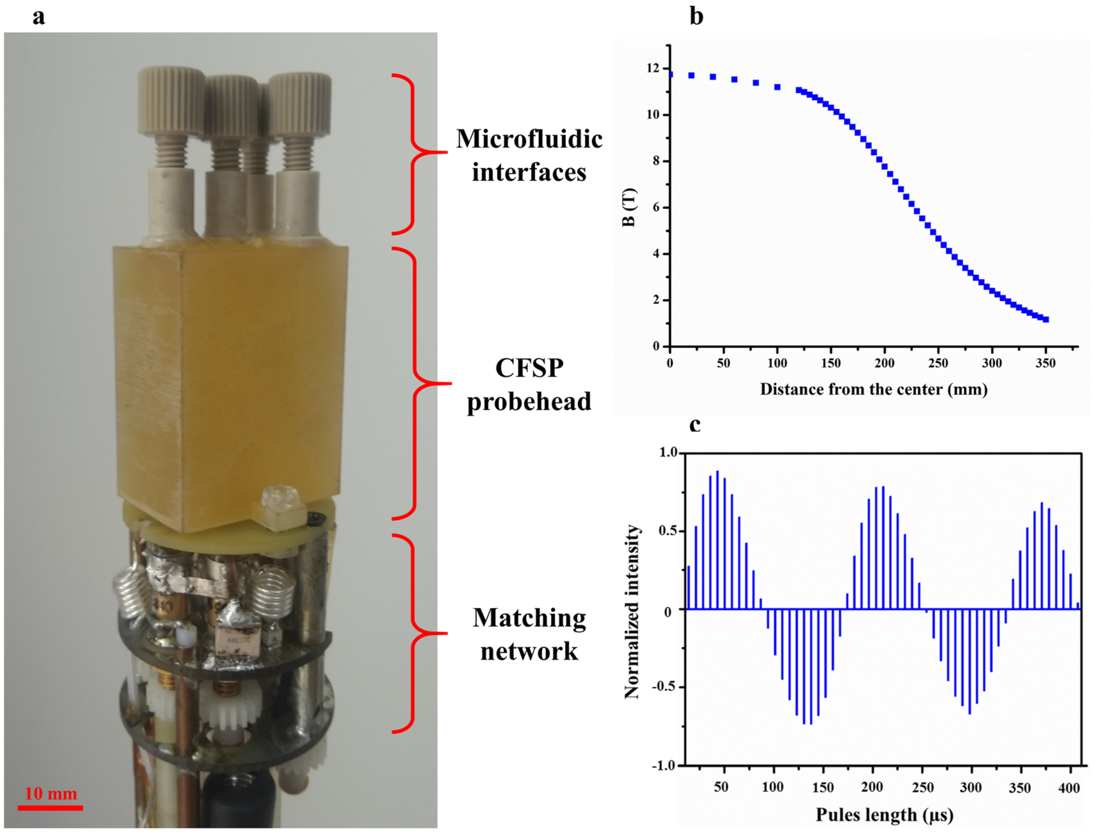

Supplementary Figure 16: The CFSP probe, measure axial field map of NMR system and nutation experimental performance. a The CFSP probe mainly consists of microfluidic interfaces, CFSP probehead, matching network and probehead support. **b** Axial field map of 11.7 T high-field magnet. **c** Experimental B1 field homogeneity tests of CFSP probe. In nutation experiment of the CFSP probe, a 90° pulse length of 9.1 μs was obtained, while the 810°/90° signal ratio was 0.85 indicating a highly homogeneous B_1 field. Source data are provided as a Source Data file.

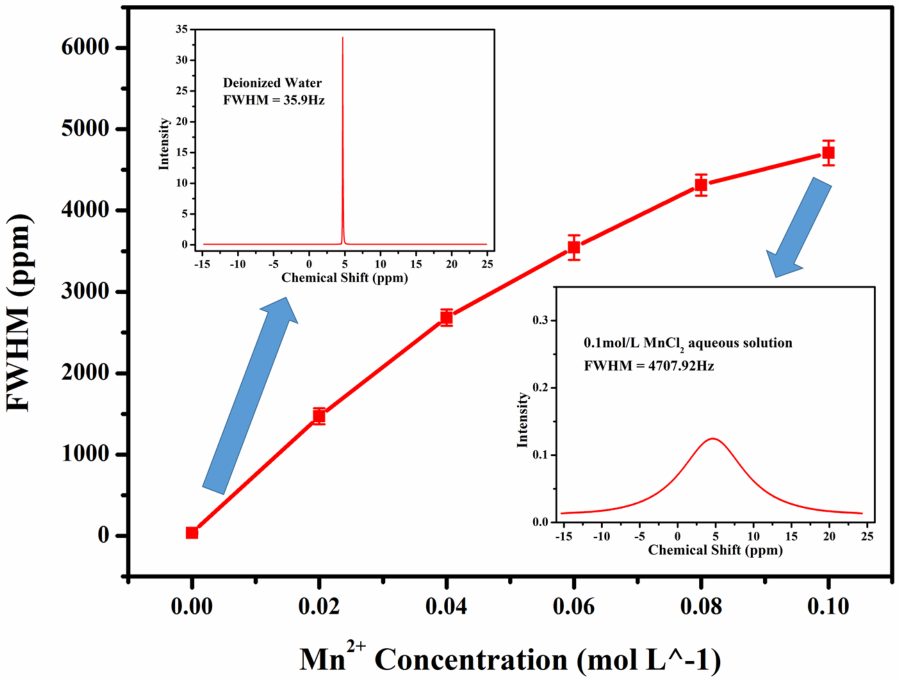

Supplementary Figure 17: The spectral FWHM measurement results of deionized water with different manganese ion concentrations. The full width at half maximum (FWHM) of the spectra deteriorates rapidly with the increase of the concentration of paramagnetic ions (75 kHz M^-1). Data represent means ± s.d. from three independent experiments. Source data are provided as a Source Data file.

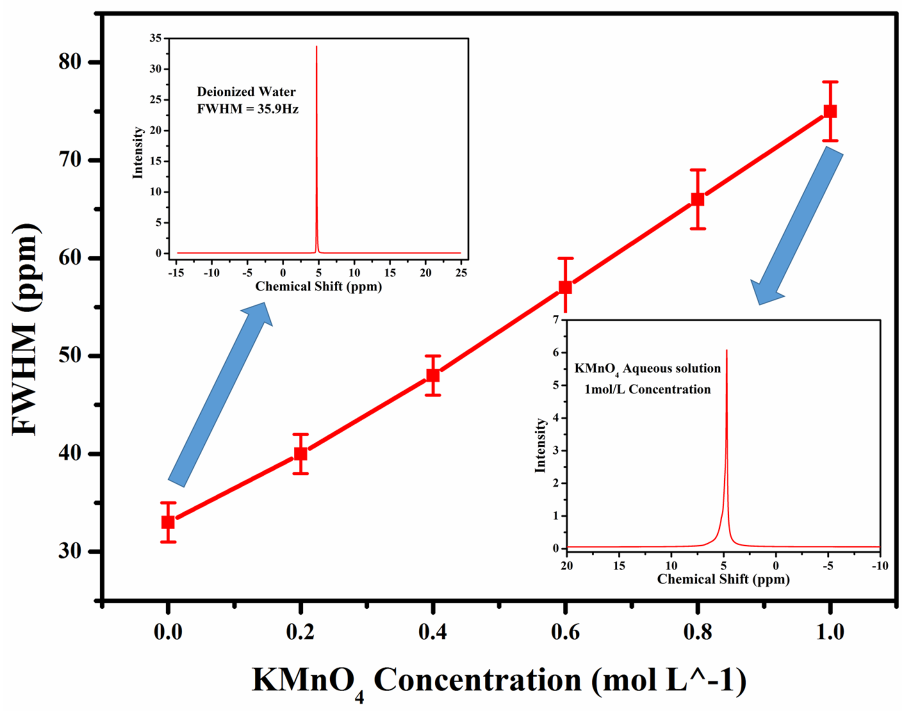

Supplementary Figure 18: The effect of potassium permanganate on the FWHM of the spectra. The spectra of potassium permanganate solution with varies concentration has a little broadening rate of spectral FWHM (30 Hz M^-1), indicating

that it has negligible effect on the paramagnetic spread of spectra. Data represent means ± s.d. from three independent experiments. Source data are provided as a Source Data file.

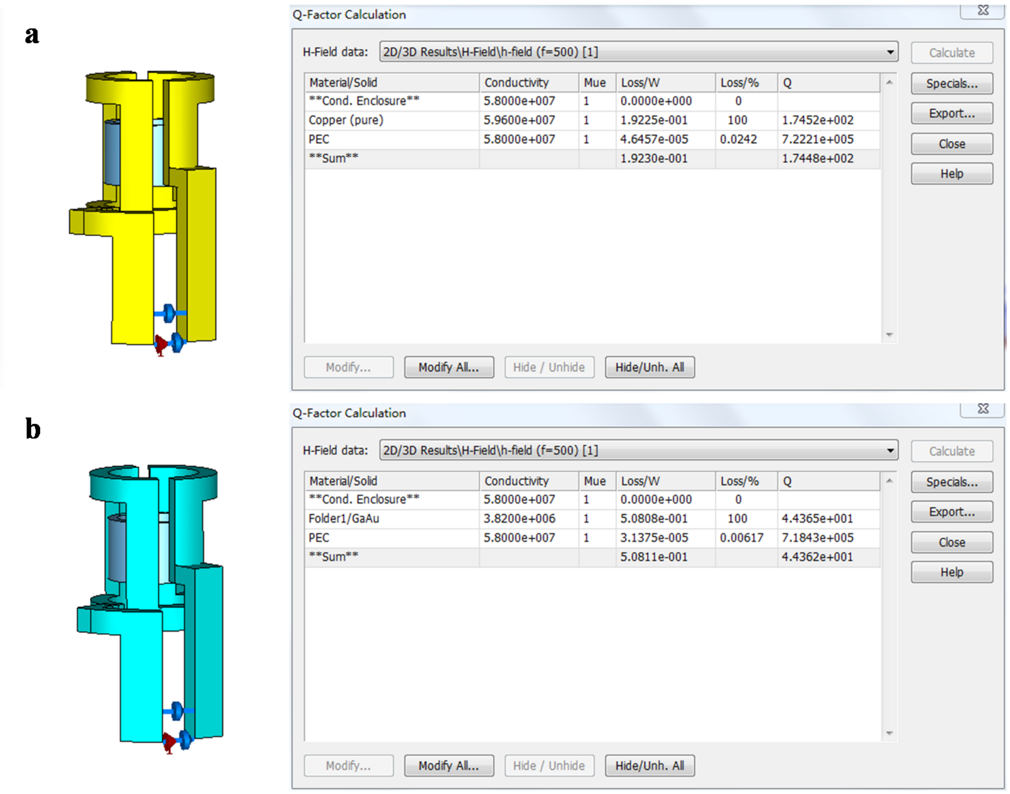

Supplementary Figure 19: CST simulation and Q factor calculation of saddle coil with different materials. The Q factor of saddle coil with copper (**a**) is about 174, while the Q factor of saddle coil with LM paste (**b**) is 44.

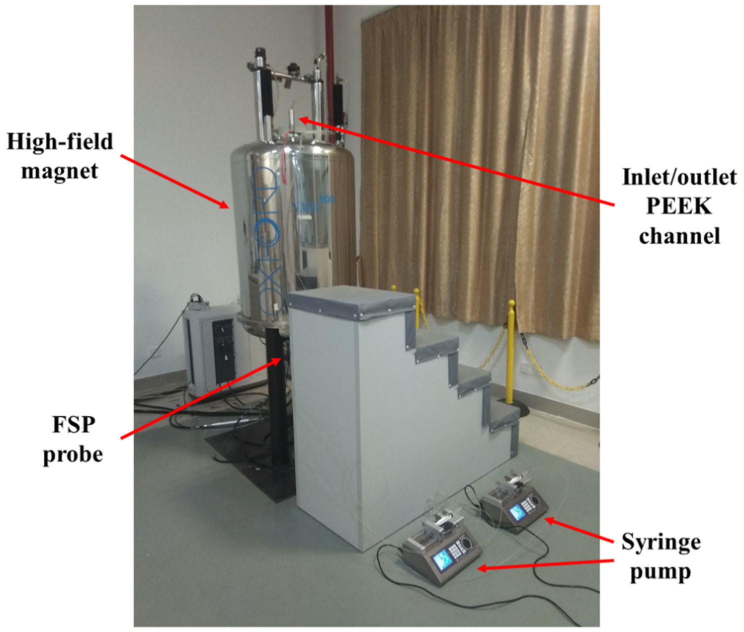

Supplementary Figure 20: Representation of the *in situ* CFSP experiment setup. Devices that may be disturbed by strong magnetic fields are placed outside the 5 Gaussian safety line.

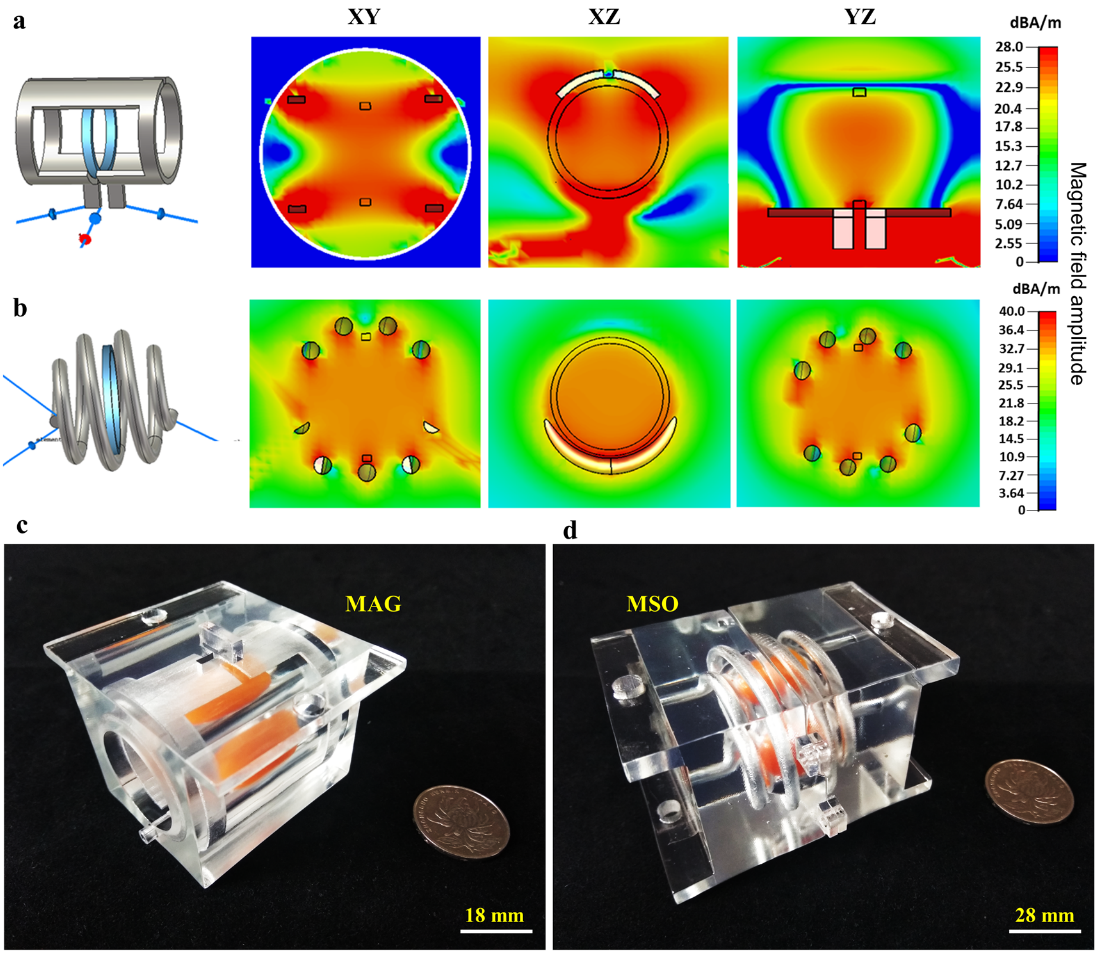

Supplementary Figure 21: Simulation and fabrication of 3D-printed MRI probehead. RF field simulations of modified Alderman-Grant (**a**) and modified saddle (**b**) resonant coils. Simulations were performed at a frequency of 300 MHz. Modified Alderman-Grant probehead (**c**) and modified saddle probehead (**d**) with a kumquat as a sample.

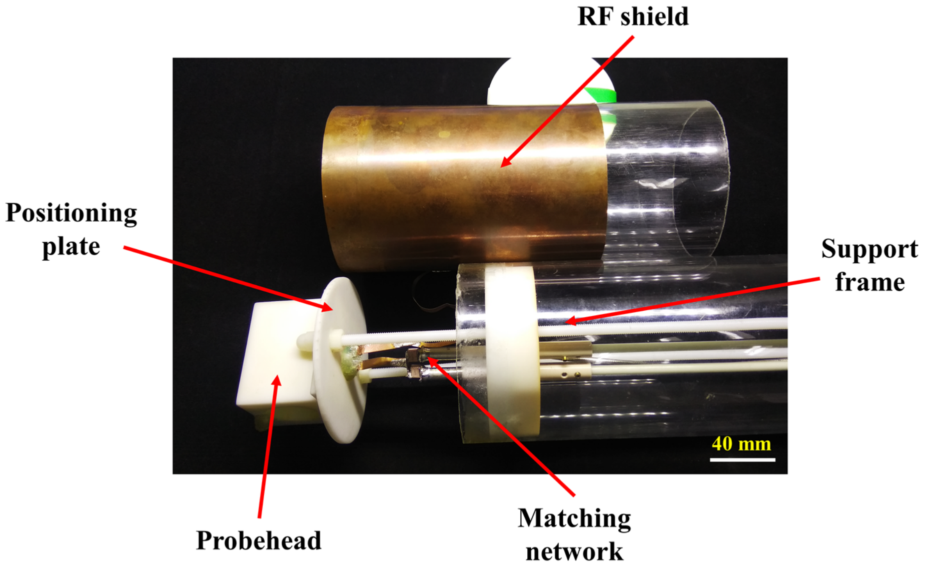

Supplementary Figure 22: The overall architecture of MRI probe. The probe mainly consists of 3D-printed probehead, positioning plate, matching network, RF shield and support frame.

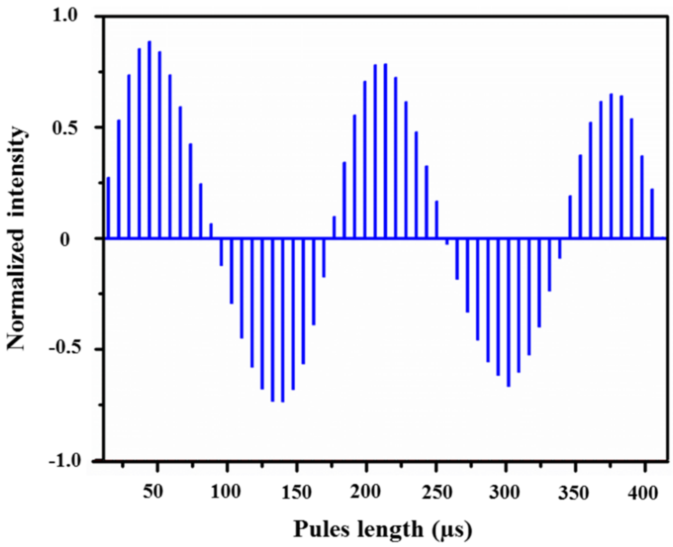

Supplementary Figure 23: Experimental B_1 field homogeneity tests of MAG probe. The 810°/90° was approximately 0.82, indicating a good actual RF field uniformity.

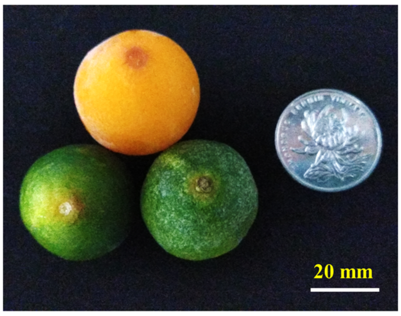

Supplementary Figure 24: Samples for MRI experiments with 3D-printed probehead. A variety of imaging samples, including kumquat and limes.

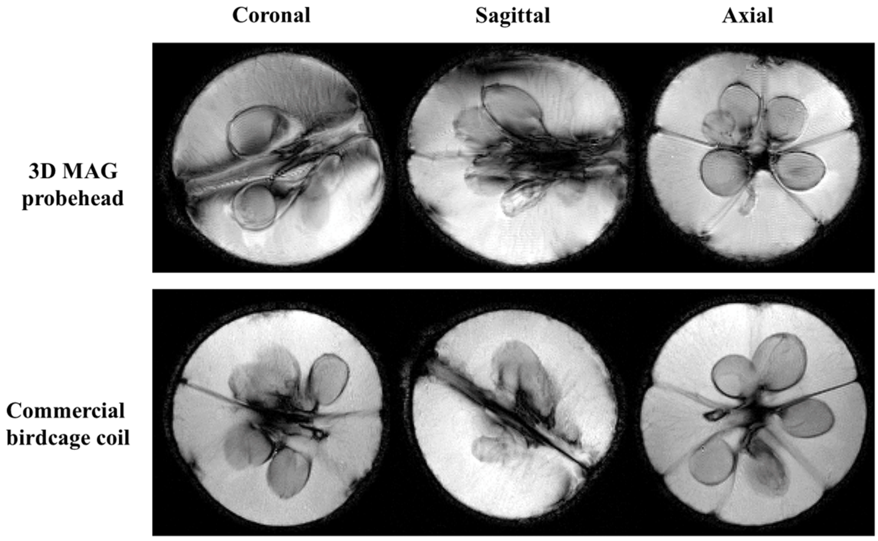

Supplementary Figure 25: MRI images of a small lime sample imaged using different probes. Compared to commercial single-channel birdcage probes, our 3D-printed probe has a higher SNR (82 dB vs. 75 dB) for small samples, giving clearer images with more detailed characteristics of the sample.

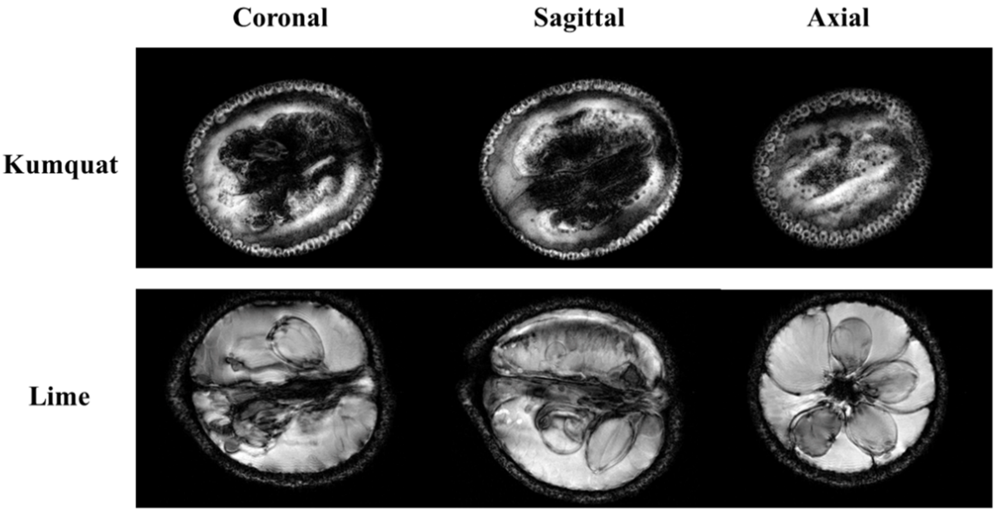

Supplementary Figure 26: MRI images of kumquat with MAG Probehead. The MAG probe also completed the construction and processed MRI experiments with kumquat and lime. Images with a similar SNR of 75 dB were acquired.

Coil structure	Dimension	Material	Unloaded *Q* factor	Working frequency	Source
**Slot tube coil (commercial)**	5.5 mm in diameter 20 mm in length	Zero magnetic susceptibility alloy	158	500MHz	Varian probe
**Double Helix Dipole coil**	7 mm in diameter 10 mm in length	Copper	150	500MHz	Reference 41
**Stripline coil**	1 mm in length 0.5mm in width	Copper	80-100	600MHz	Reference 42
**Solenoid coil**	0.338 mm in diameter 1 mm in length	Polyurethane-coated copper wire	26	300MHz	Reference 43
**MRI surface coil**	25×25 mm^2 1 mm in wire width	Sliver paste	12.5	400MHz	Reference 44
**MRI solenoidal microcoil**	0.7 mm in diameter 1.5 mm in length	Insulated gold wire	46	400MHz	Reference 45
**Our LM saddle coil**	**3 mm in diameter 4.98 mm in length**	**Gallium based liquid metal paste**	**43.5**	**500MHz**	**--**

Supplementary Table 1: Comparison of several MR probes. Structure, material and performance of several NMR and small-scale MRI coil were compared, including our 3D-printed SAP probehead.

Supplementary Methods

3D printed model post-processing. The 3D-printed probeheads are subject to few post-processing procedures, the contents of which vary depending on the selected printing method. As for FDM, the printed bodies are immersed in mineral oil bath at 80℃ to dissolve the sacrificial materials by the ultrasonic cleaning after printing. Residual oils on the surface of 3D-printed prototypes are removed in hot water bath. Due to their sensitivity to UV laser, the photopolymer resins are photo chemically solidified quickly under irradiation and form the probehead layer by layer during SLA printing. The 3D-printed models must be washed with a solvent (e.g. isopropanol) to clean residual resin off their surfaces and internal channels. The supporting structures added to ensure successful printing are also manually removed after cleaning, and the models are placed under natural light for several hours subsequently to complete the secondary curing.

3D printing building material measurement. Using Vector Network Analyzer, we measured scattering parameters of a parallel RLC resonator test fixture (Supplementary Fig. 1a), which used the materials under test (MUT) as dielectrics of parallel plate capacitor. Equivalent circuit is shown in the Supplementary Fig. 1b. Several kinds of 3D printing building materials were constructed in the form of rectangular cuboids with the size of 25 mm × 20 mm × 5 mm as the filling dielectric materials (Supplementary Fig. 2c). Polytetrafluoroethylene (PTFE) with a known permittivity ($\varepsilon_r = 2.5$, $\tan\delta = 0.0002$) was used to validate the accuracy of the method and calculate the electrical performance parameters of MUT. The transmission coefficients of the resonator circuit are measured in a range of frequencies containing its resonant frequency (300 MHz and 500 MHz) using VNA.

LM optimization. We first incorporated silver microparticles (AgMPs) with a diameter of 10 μm as hybrid particles because of their high conductivity compared to that of other metals. However, we found that after adding AgMPs, the resistance increased (Supplementary Fig. 5). This increase may be due to the additional oxidation of silver microparticles and gallium, and the introduced voids during the mixing process. We then chose gold microparticles (AuMPs) as mixed metal instead to offset the increase in resistivity resulting from oxide buildup due to their stability and relatively high conductivity.

Electric performance measurement of LM pastes. To complete the measurement of different kinds of LM pastes in more precision, electrical performance testing models made from alternative conductive LM materials were fabricated in a 3D printing mold, on top of which a 45 mm × 3 mm × 2 mm I-shaped groove was printed. After a smooth cavity filling, extrusion and solidification, LM pastes were stereotyped as the shape of the groove. We measured electrical conductivity of LM pastes by 4-wire (Kelvin) resistance measurement method with GPD-2303S (GWinstek Electronics

Industrial Co., Ltd., China) as DC power supply and 34410A $6^1/_2$ digit multimeter (Agilent Technologies Inc., USA) as voltmeter. Five models of each LM material are fabricated to minimize the errors of production and measurement. Using video optical microscopy (FT-QT500A, Shenzhen Font Technology Co., Ltd., China), an area of 300 μm × 300 μm is imaged at the mold surface using an image processing software.

SAP design and postprocess. A one-turn saddle-shaped coil channel with 4.98 mm in height, 3 mm in diameter and 0.78 mm × 0.6 mm wire cross-sectional area, is constructed for instance. Hollow micro channels and sample chambers are designed in the cube substrate structures to be filled later with LM and samples respectively to form MR coils and detection regions. To facilitate the LM infusion process, we designed a specialized injection hole (as inlet), with an internal diameter of 600 μm, in opposite side of saddle coil pins (as outlet). The LM coil was connected to a matching network via copper plates as feedlines, with custom designed inserting channels. We proposed the RF circuit interfaces design with different wire cross-sectional areas (0.3 mm × 0.6 mm and 1.2 mm × 1 mm, respectively) in the LM filled micro channels. The narrow exits of micro channels facilitate the seal of coil pins after inserting thin copper strips for circuit connections, while the wide inside parts made inserted copper strips completely immersed in LM, guaranteeing well electrical contact conductivity and stability for connection between the conductive LM coil and RF circuit. Sample inlet/outlet was designed as prominent structures to match lids and microfluidic interfaces. Two smooth transition structures were also designed to connect detection region with sample inlet/outlet chambers. This design enlarges the sample volume in the detection region, therefore improves filling factor and sensitivity of coils. A series of capacitors with a capacitance range between 1 and 19 pF are used for optimized tuning and matching. The chip capacitors and the variable capacitors were purchased from Voltronics Corp.

Flow rate setting. The ethanol and potassium permanganate water solution were mixed inside the probehead at the Y-junction inside the model. The channel length is sufficient to allow the two reactants to mix and react adequately even at the highest flow rate (100 μL min^-1 for each channel) in this work. To monitor the oxidizing reaction process by continuous-flow, the residence time at the NMR detecting area should be tuned to the time scale of the process. If the flow rate is too high, the excited sample will flow out of the detection area before the NMR signal is completely sampled, resulting in a decrease in spectral resolution. We did not observe such an effect with the settings used in this paper. After the sample is fully filled with the channel, the flow rate remains stable and consistent with the injection pump setting.

***In situ* CFSP experiment setup.** To reduce dead volume from pump to probehead, we use polyether ether ketone (PEEK) tubes with inner diameter (ID) of 0.025 inch to connect the pump and inlet channels. The outlet flow path uses a thicker PEEK tube with ID of 0.04 inch to prevent blockage of the passage by solid products and reduce

the back pressure of probehead. Commercially available PEEK coned nano ports were used to connect tubes and probehead. The total volume of the fluids inside the fluidic system was experimentally measured to be around 800 μL.

MRI probehead assembly and experiment. To enhance the reliability of the electrical connection with RF coils, the copper strips were prewetted with the LM pastes. Connecting cables and variable capacitors, which are used for matching/tuning the coil, were soldered onto the copper strips as discussed (Supplementary Fig. 17). The specially designed probe bracket structure steadily supported the probeheads and matching network. The MAG and MSO probes were first assembled and tested offline using a vector network analyzer to measure the unloaded quality factors (equals to 50 and 55, respectively). The B_1 field homogeneity was assessed by processing nutation experiments (Supplementary Fig. 18). A PdWI weighted gradient echo pulse sequence was used for MRI. The echo time was set to 5 ms, the repetition time was 100 ms, nt=16, the field of view was 45 mm × 45 mm with a resolution of 256 × 256 lines, and the slice thickness was 2 mm.

Supplementary Note 1

Magnetic resonance imaging using 3D-printed probehead. MRI is an important field in the application of magnetic resonance techniques and has been widely used in many fields for years. According to the detection demands of different application scenarios, customized MRI coils conformed to the shape of samples are necessary to obtain clear structural features of tiny structures in objects, especially for small-volume samples. We present here a practical stratergy for this requirement through 3D-printed integrative MRI probeheads.

According to the large size and the required noncapacitive structure of 3D-printed probehead coils noted above, the modified AG structure is a relatively good selection for MRI experiments. In the modified AG coil, the current is distributed over the surface of a curved copper foil instead of flowing along a long path of thin wire, and the length of the current pathway is shorter, reducing the total amount of coil inductance. This configuration is hence more suitable for high-frequency MRI without introducing additional thermal noise and extra heating of the samples. A modified solenoid coil was also designed to achieve the imaging of small circular samples, such as kumquats or limes. As a bicone more closely conforms to the anatomical cross section of these fruits, better filling factors and therefore improved SNRs can be achieved by using micro-bicone-shaped modified solenoid coils. These two coils were simulated using CST Microwave Studio to work at 300 MHz, the resonant frequency of protons in the Varian 7T animal MRI system (Supplementary Fig. 21a and 21b). For the modified AG coil with a 50 mm height, 30 mm inner diameter and 90° window angle, an inferior RF field uniformity was obtained compared with that of the modified solenoid coil with 4 turns in total, an inner spherical-like diameter of 30 mm, a 45 mm height and a wire diameter of 2 mm. Clearly, the equivalent wire lengths of both coils are below the acceptable limitation of $\lambda/10$, which means that they will perform better than conventional solenoids or AG coils with the same dimensions. Therefore, we utilized the modified solenoid coil to image small structures in MRI experiments, while the modified AG coil was used for the detection of large samples.Probeheads were printed and fabricated with the design size described above. Due to the large area of the inner channel structure of MAG, we designed two injection holes on opposite sides of the probehead model to ensure the metal filling integrity and accuracy of the coil (Supplementary Fig. 21c). Two additional copper strips were used as feedlines between the two cone-shaped coils of the MSO, and 4 fastening structures were designed to enhance the stability of the probe after assembly, as shown in Supplementary Fig. 21d. A lime with an average diameter of ~30 mm was chosen as the subject in the MRI experiments because of its structured tissue (carpels), high content of hydrogen, and spherical shape suitable for demonstrating the conformal fit of our customized coils (Supplementary Fig. 24). Supplementary Fig. 25 shows MRI images of a lime taken by the bicone-shaped microcoil at a volume size of 20 mm × 20 mm × 25 mm. The SNR of the MRI experimental image was calculated by the following formula:

$$\mathrm{SNR} = 10\log\frac{\mathrm{mean}\left(\mathrm{Signal}^2\right)}{\mathrm{var}\left(\mathrm{Noise}\right)}. \quad (2)$$

The measured SNR of the 3D-printed MSO probehead (82 dB) was higher than that of commercial probes (75 dB). The commercial single-input birdcage probe (TM-DF-7R0-002, Time Medical (Jiangsu) Co., Ltd) used for experimental comparisons has a sample cavity with inner diameter of 65 mm and unloaded Q factor of 40 approximately. As expected, a larger fill factor provided a higher SNR. The details of the inner structure of the lime can be clearly observed. The performance of the MAG probehead was also evaluated by MRI experiments with lime and kumquat samples and also showed good imaging results (Supplementary Fig. 26).
